# Cell type‐specific regulation of m^6^A modified RNAs in the aging *Drosophila* brain

**DOI:** 10.1111/acel.14076

**Published:** 2024-01-11

**Authors:** Alexandra E. Perlegos, China N. Byrns, Nancy M. Bonini

**Affiliations:** ^1^ Neuroscience Graduate Group, Perelman School of Medicine University of Pennsylvania Philadelphia Pennsylvania USA; ^2^ Department of Biology University of Pennsylvania Philadelphia Pennsylvania USA; ^3^ Medical Scientist Training Program, Perelman School of Medicine University of Pennsylvania Philadelphia Pennsylvania USA

**Keywords:** aging, Alzheimer's disease, *Drosophila*, epitranscriptomics, m^6^A, neurodegeneration

## Abstract

The aging brain is highly vulnerable to cellular stress, and neurons employ numerous mechanisms to combat neurotoxic proteins and promote healthy brain aging. The RNA modification m^6^A is highly enriched in the *Drosophila* brain and is critical for the acute heat stress response of the brain. Here we examine m^6^A in the fly brain with the chronic stresses of aging and degenerative disease. m^6^A levels dynamically increased with both age and disease in the brain, marking integral neuronal identity and signaling pathway transcripts that decline in level with age and disease. Unexpectedly, there is opposing impact of m^6^A transcripts in neurons versus glia, which conferred different outcomes on animal health span upon *Mettl3* knockdown to reduce m^6^A: whereas *Mettl3* function is normally beneficial to neurons, it is deleterious to glia. Moreover, knockdown of *Mettl3* in glial tauopathy reduced tau pathology and increased animal survival. These findings provide mechanistic insight into regulation of m^6^A modified transcripts with age and disease, highlighting an overall beneficial function of *Mettl3* in neurons in response to chronic stresses, versus a deleterious impact in glia.

## Graphical abstract

m^6^A levels increase with age in the brain and in the brains of animals expressing human Aβ42 in neurons. Knockdown of *Mettl3* in neurons decreases longevity and but increases translation efficiency by TRAP assay, and increases DNA damage. Knockdown of *Mettl3* in glial cells increases longevity and decreases translation efficiency of m^6^A modified transcripts.

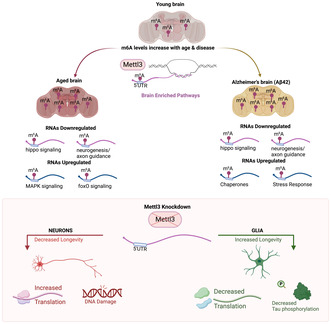

**Abbreviations**
APP/PS1: amyloid precursor protein/presenilin 1
ER: endoplasmic reticulum
FACS: fluorescence‐activated cell sorting
h: hours
HR: homologous recombination
MAPK: mitogen‐activated protein kinase
min: minutes
m^6^A‐IP: m^6^A immunoprecipitation
PCA: principal component analysis
RNA‐seq: RNA sequencing
TBI: traumatic brain injury
TCA: tricarboxylic acid
TGF‐beta: transforming growth factor beta
TRAP: translating ribosome affinity purification

## 1 INTRODUCTION

Aging increases the brain's susceptibility to neurodegeneration and cognitive decline, making it crucial to understand mechanisms that impact the aging brain. Key factors that modulate brain health and protein neurotoxicity include changes in transcriptional silencing and activation, protein translation, and the stress response. Dysregulation of glia, which provide metabolic and structural support for neurons, is also a key hallmark of aging (Zuchero & Barres, 2015). The aging brain is characterized by increased cellular stress markers such as DNA damage and oxidative stress, decreased mitochondrial function, and increased protein misfolding (Haigis & Yankner, 2010). Overall, aging and the onset of neurodegenerative disease reflects an imbalance of damage caused by cellular stress and chronic activation of stress response pathways aiming to help. Insights gained from studying the basic biology of aging have identified pathways linked to lifespan extension including calorie restriction and lowering of metabolic rate, upregulated stress response chaperones, restoration of mitochondrial dysfunction, and changes in epigenetic modifications (Bonini, 2002; Bordone & Guarente, 2005; Haigis & Yankner, 2010; Kennerdell et al., 2018; Ruggiero et al., 2008; Sen et al., 2016).

RNA modifications have been implicated as fundamental regulators of gene expression. m^6^A, one of the most abundant RNA modifications found on eukaryotic mRNA, has been linked to diverse functions including cell differentiation, mRNA stability, splicing, secondary structure, translation efficiency, and chromatin remodeling (Fu et al., 2014; Liu et al., 2017; Liu, Dou, et al., 2020; Wang et al., 2014; Xiao et al., 2016; Zhao et al., 2017). m^6^A is dynamically regulated by a methyltransferase complex consisting of the catalytically active component METTL3, and downstream nuclear and cytoplasmic reader proteins that govern the fate of m^6^A modified RNAs (Lence et al., 2017; Shi et al., 2019; Yang et al., 2018). The levels of m^6^A are highest in the brain, where it modulates transcripts important for many biological processes such as synaptic plasticity (Shi et al., 2018), learning and memory (Kan et al., 2021; Shi et al., 2018; Walters et al., 2017), development (Ma et al., 2018; Wang et al., 2018), neurogenesis (Li et al., 2017; Yoon et al., 2017), gliogenesis (Cui et al., 2017; Xu et al., 2020) and subcellular localization of transcripts (Loedige et al., 2023). Altered levels of m^6^A and its regulatory complex proteins have been implicated in the pathogenesis of neurological disorders including Alzheimer's disease (Li et al., 2018), Parkinson's disease (Chen et al., 2019), amyotrophic lateral sclerosis (McMillan et al., 2023), and glioblastoma (Cui et al., 2017; Li et al., 2019; Zhang et al., 2017). Understanding the role of m^6^A in the normal regulation of brain homeostasis, therefore, will provide valuable insights into mechanisms connecting aging, stress, and disease.

Under periods of stress, RNAs are transported for prompt degradation or for selective translation, and m^6^A regulation of RNA processing is heightened. Studies in mammalian cells in vitro show that the regulation of m^6^A is critical for acute cellular stress conditions including UV‐induced DNA damage and heat shock (Ji et al., 2021; Xiang et al., 2017; Yu et al., 2021; Zhou et al., 2015). Additionally, studies conducted in vivo have shown that m^6^A increases upon restraint stress in mice, and m^6^A is necessary for axon regeneration after injury (Engel et al., 2018; Weng et al., 2018). Furthermore, m^6^A modified RNAs and reader proteins are concentrated in stress granules that form in response to cellular stress (Anders et al., 2018; Fu & Zhuang, 2020).

*Drosophila* has revealed that m^6^A plays a critical role in vivo in regulating the brain's acute heat shock response (Perlegos et al., 2022), which is important for handling the stress and promoting recovery (Leak, 2014). Here, we investigate the role of m^6^A in the brain during chronic stresses of aging and progressive degenerative disease. Upon aging and disease, m^6^A levels increase on target transcripts of signaling pathways and neurogenesis, which are pathways that normally become downregulated in the brain with age and disease. Moreover, knockdown of m^6^A methyltransferase *Mettl3* in neurons versus glial cells yields a markedly different impact on the DNA damage response, translation efficiency of m^6^A marked transcripts, and animal health span. In addition, knockdown of *Mettl3* in glia reduced pathological tau burden and dramatically extended lifespan in a fly model of glial tauopathy. Overall, these studies indicate that m^6^A RNA modification plays a critical role in the brain to impact chronic stresses of aging and disease, with a notably different impact based on cell type.

## 2 RESULTS

### 2.1 m^6^A levels increase in the 5' UTR of transcripts in the aging brain

**FIGURE 1:**
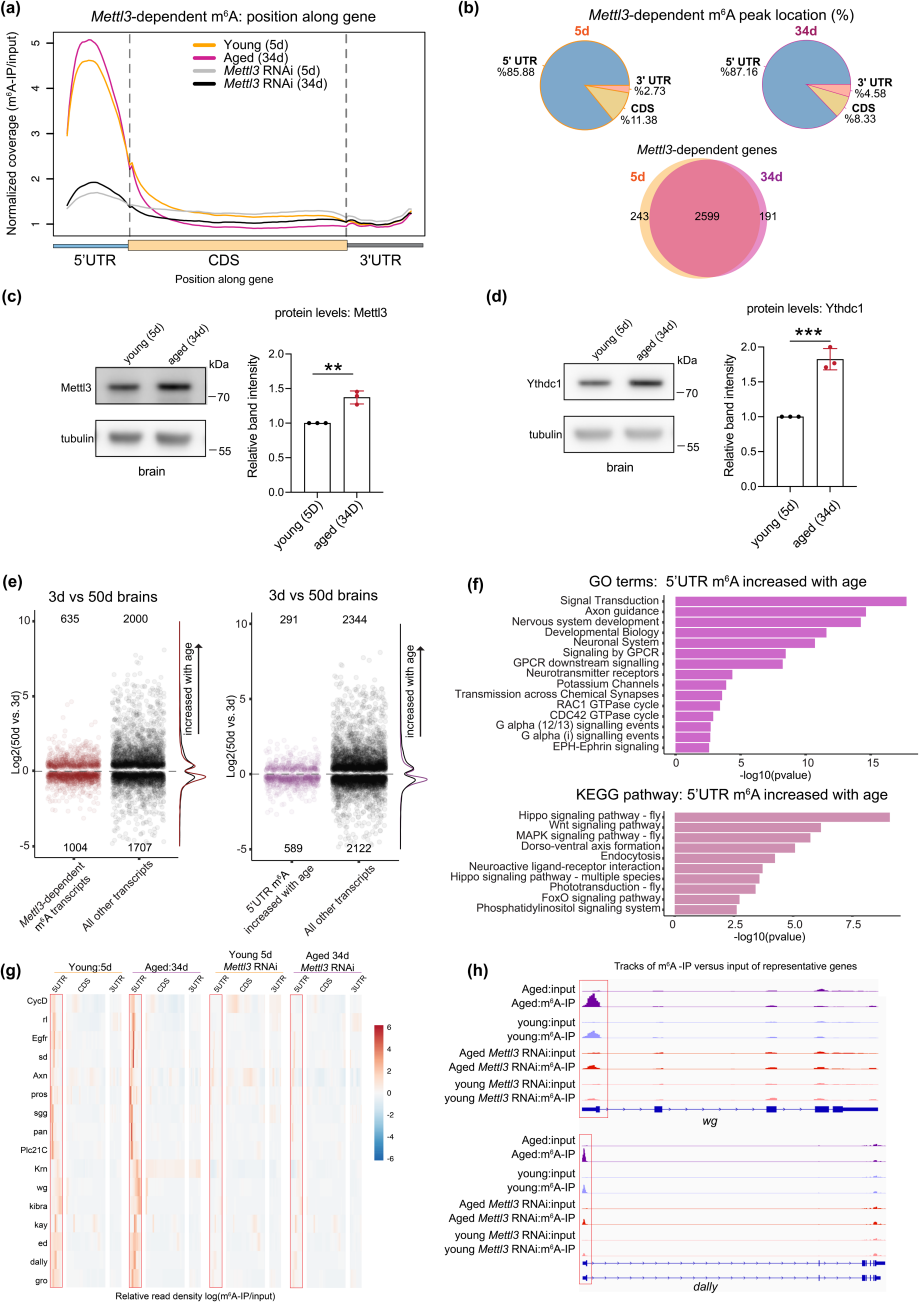
m^6^A increases in the 5' UTR in the brain with age. (a) Normalized read coverage plot of m^6^A‐IP/input on polyA+ transcripts across the 5' UTR, CDS, and 3' UTR of *Mettl3*‐dependent transcripts. m^6^A‐IP sequencing in 5d and 34d conditions from control RNAi and *Mettl3* RNAi fly heads (*da*Gal4 > mCherry RNAi; *da*Gal4 > *Mettl3* RNAi). *Mettl3* RNAi samples show a loss of m^6^A primarily in the 5' UTR. (b) Transcript location of *Mettl3*‐dependent m^6^A at 5d and 34d. m^6^A transcripts at 5d and 34d show a 93% overlap. (c) Protein levels of Mettl3 with aged brains (5d vs. 34d), **p < 0.01, *p* = 0.0024, Student's *t*‐test. (d) Protein levels of Ythdc1 with aged brains (5d vs. 34d), ***p < 0.001, *p* = 0.0007, Student's *t*‐test. (e) Plot of significantly differentially expressed genes *p*_adj_ < 0.05 of control (*w* ^*1118*^) brains with age (50d vs. 3d). Positive logFC indicates an increase in transcript level with age. *Mettl3*‐dependent m^6^A transcripts (red), all other non‐m^6^A transcripts (black), transcripts with increased 5' UTR m^6^A with age (purple). (f) GO and KEGG pathway enrichment of transcripts with increased 5' UTR m^6^A methylation with age (34d vs. 5d). (g) Heat map of m^6^A enrichment on signaling pathway (KEGG) transcripts with increased 5' UTR m^6^A with age. Shown are Control RNAi and *Mettl3* RNAi m^6^A enrichment in 5d and 34d conditions. m^6^A enrichment presented as log (m^6^A‐IP divided by the input control). Heat map displays *z*‐score values scaled by row, with each gene relative to itself and relative across all six boxes. Segmented into 5' UTR, CDS, and 3' UTR. (h) Genome browser tracks of m^6^A locations for example genes *dally* and *wg* at 5d and 34d time points from control and *Mettl3* RNAi.

To assess m^6^A modification with age we conducted m^6^A‐IP sequencing analysis from 34d versus 5d *Drosophila* head samples with and without *Mettl3* knockdown (Data S1). Consistent with previous work (Kan et al., 2021; Perlegos et al., 2022), m^6^A‐IP sequencing showed that m^6^A is enriched in the 5' UTR of transcripts and dependent on the methyltransferase *Mettl3* (Figure 1a,b and Figure S1a). 5' UTR m^6^A enrichment is unique to *Drosophila*, as mammalian m^6^A is primarily in the 3' UTR (Meyer et al., 2012). We used RADAR differential methylation analysis to define *Mettl3*‐dependent m^6^A modified transcripts, referred to here as m^6^A transcripts (Data S2). Global m^6^A levels were strikingly increased with age (Figure 1a), with 49% of all m^6^A transcripts having significantly increased 5' UTR m^6^A (1455 of a total of 3033 m^6^A transcripts) upon aging (Data S2). m^6^A transcripts significantly overlapped at 34d and 5d, indicating that the same transcripts were marked at both timepoints, but with increased levels of m^6^A at 34d (Figure 1b). To further understand these changes, we examined the levels of m^6^A methyltransferase and the Ythdc1 reader protein in young versus aged brains. Mettl3 protein levels increased ~1.4 fold (Figure 1c), which may in part account for the overall increase of m^6^A on transcripts with age. The levels of nuclear reader protein Ythdc1 were also significantly increased with age (~1.8 fold, Figure 1d). Overall, m^6^A methylation was increased with age on a subset of transcripts in the brain, in association with changes in methyltransferase and reader protein abundance.

We then investigated if m^6^A transcripts were differentially expressed with age in the brain (Srinivasan et al., 2022). The aging brain has distinct transcriptional changes, such as upregulation of transcripts of the stress‐response, oxidative stress and mitochondrial damage, and overall transcriptional decline due to altered RNA processing (Gemma et al., 2007; Haigis & Yankner, 2010; Ham & Lee, 2020; Sen et al., 2016). Non‐m^6^A marked genes expressed in the brain showed an equal distribution of up and down regulation with age; by contrast, m^6^A marked transcripts were more downregulated in both mid‐age (20d) and advanced age (50d) brains (Figure 1e and Figure S1b). Similarly, transcripts with increased 5' UTR m^6^A were more downregulated with age (Figure 1e and Figure S1b,c).

To further define the m^6^A marked transcripts with age, we compared these to the total brain transcriptional response. Examining all genes in the brain, those upregulated with age were enriched for proteasome, nonsense‐mediated decay, and toll signaling, whereas downregulated pathways were enriched for oxidative phosphorylation, TCA cycle, and phosphatidylinositol signaling (Figure S1d,e; Data S3). Only a subset of all genes expressed in the fly brain are marked by m^6^A (~14%) (Perlegos et al., 2022), yet m^6^A transcripts represented ~30% of differentially expressed genes upon aging (Figure 1e and Data S4). Examining all transcripts with increased m^6^A with age showed enrichment for pathways critical in the function of neurons (signaling and neurogenesis pathways) (Figure 1f–h). Further sub‐setting transcripts with increased 5' UTR m^6^A and downregulated with age showed enrichment for axon guidance, Hippo signaling, glycerophospholipid metabolism, and phosphatidylinositol signaling (Figure S1f); these marked pathways are likely less functional with age. Interestingly, a smaller set of transcripts with increased m^6^A that were instead upregulated with age were enriched for pathways known to impact lifespan, including MAPK, Notch, foxO, and TGF‐beta signaling, highlighting a subset of signaling pathways oppositely regulated by m^6^A with age (Figure S1g). These findings underscore two opposite avenues of m^6^A regulation in the brain with age, indicating a complex interplay between m^6^A and age‐related gene expression changes.

### 2.2 Alzheimer's disease model shows increased 5' UTR m^6^A

**FIGURE 2:**
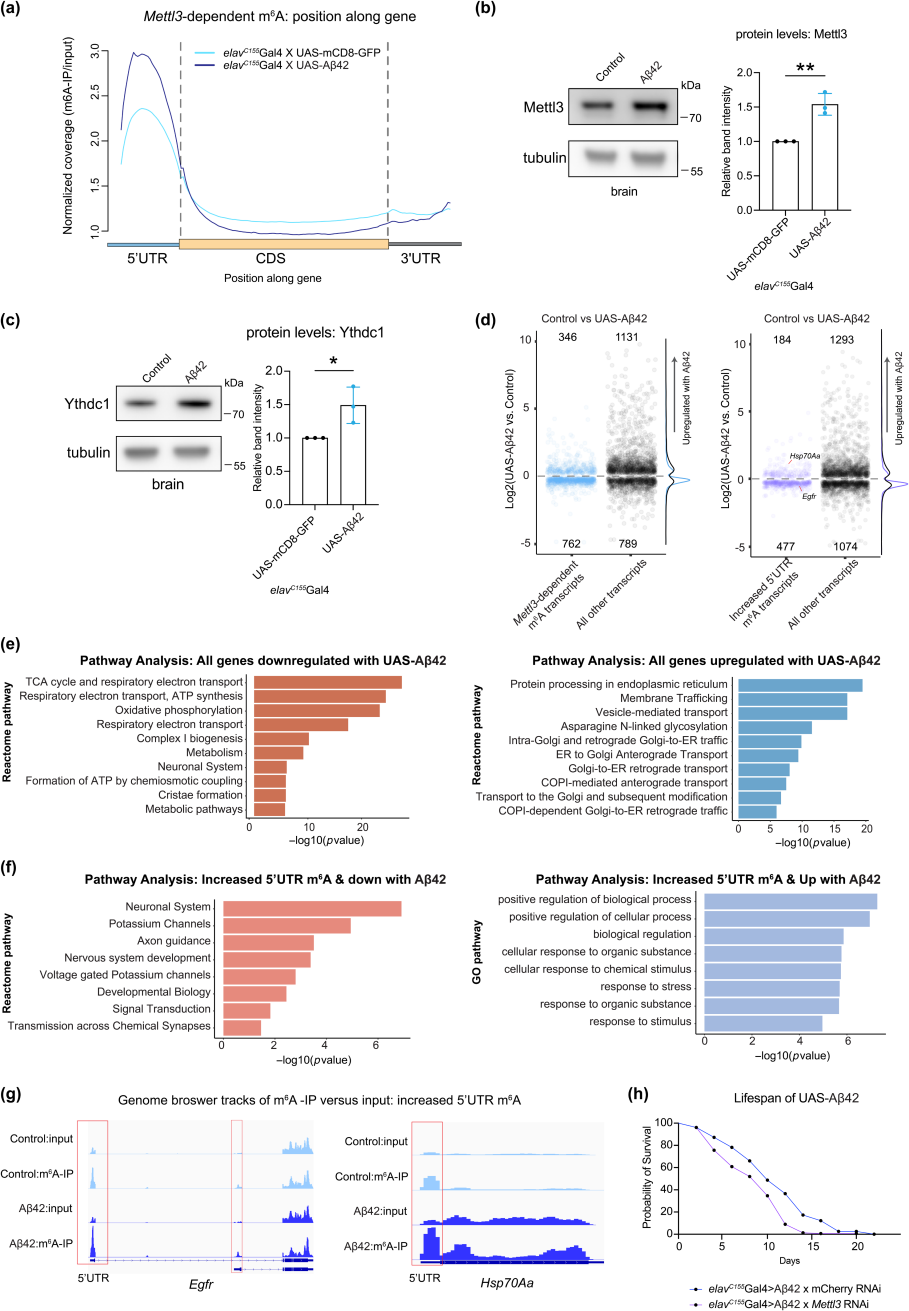
Alzheimer's disease model shows increased 5' UTR m^6^A. (a) Normalized read coverage plot of m^6^A‐IP/input on polyA+ transcripts in the 5' UTR, CDS, and 3' UTR of m^6^A transcripts. m^6^A‐IP sequencing in control (6d) and Aβ42 (6d) conditions. (*elav* ^C155^Gal4 > UAS‐mCD8‐GFP; *elav* ^C155^Gal4 > UAS‐Aβ42). Aβ42 head samples show an increase of m^6^A primarily in the 5' UTR. (b) Protein levels of Mettl3 in Aβ42 brains, **p < 0.01, *p* = 0.0042, Student's *t*‐test. (c) Protein levels of Ythdc1 in Aβ42 brains, *p < 0.05, *p* = 0.0355, Student's *t*‐test. (d) RNA seq from 6d brains of *elav* ^C155^Gal4 > UAS‐mCD8‐GFP and *elav* ^C155^Gal4 > UAS‐Aβ42. Plot of significantly differentially expressed genes of control versus Aβ42 brains. Positive logFC indicates an increase in transcript level with Aβ42 expression. m^6^A transcripts (blue), m^6^A genes with 5' UTR increased peaks (purple), all other differentially expressed genes (black). (e) Left: Pathway analysis of all genes downregulated in UAS‐Aβ42 brains. Right: Pathway analysis of all genes upregulated in UAS‐Aβ42 brains. (f) Left: Pathway analysis of all genes downregulated in UAS‐Aβ42 brains with increased 5' UTR m^6^A. Right: Pathway analysis of all genes upregulated in UAS‐Aβ42 brains with increased 5' UTR m^6^A. (g) Example genome browser tracks of increased 5' UTR m^6^A locations for genes *Hsp70Aa* and *Egfr* from control and UAS‐Aβ42. (h) Lifespan of animals expressing control RNAi or *Mettl3* RNAi in neurons (*elav* ^C155^Gal4 > mCherry RNAi vs. *elav* ^C155^Gal4 > *Mettl3* RNAi). 29°C, *n* = 156, *n* = 156, ****p < 0.0001, Log‐rank test.

As aging is a major risk factor for degenerative disease, we extended these studies to a *Drosophila* model of Alzheimer's disease that expresses toxic human Aβ42 in neurons (Iijima et al., 2004). m^6^A‐IP sequencing (6d, *elav* ^C155^ > UAS‐Aβ42 vs. *elav* ^C155^ > UAS‐mCD8GFP) showed dramatically increased levels of 5' UTR m^6^A and a significant overlap (57%) with m^6^A transcripts from the aging m^6^A‐IP (Figure 2a; Figure S2a). Transcripts with increased m^6^A were enriched for signaling and neurogenesis pathways (Figure S2b), indicating similar pathways are regulated by m^6^A in Aβ42 brains and normal brain aging. To further understand the increase in m^6^A with Aβ42, we examined m^6^A methyltransferase and reader protein levels. Similar to aged brains, there were increased levels of Mettl3 protein (~1.5‐fold) and nuclear reader protein Ythdc1 (~1.5‐fold) (Figure 2b,c). These findings suggest that increased m^6^A modification and altered activity of its associated proteins may play a role in the response of the brain to Aβ42.

We then investigated how the m^6^A transcriptional response compared to the total brain Aβ42 transcriptional response. RNA‐sequencing from Aβ42 and control brains showed that m^6^A transcripts were more downregulated in disease brains compared to non‐m^6^A transcripts (Figure 2d). Overall, ~50% of all genes differentially expressed with Aβ42 were also changed with age (Figure S2c), and, similar with normal aging, m^6^A transcripts were more downregulated in disease brains, suggesting these signaling pathways likely become less functional. Transcripts upregulated with Aβ42 expression were enriched for pathways associated with ER membrane protein processing and stress, whereas downregulated pathways were enriched for oxidative phosphorylation and metabolism (Figure 2e). m^6^A transcripts downregulated in Aβ42 brains highlighted transcripts for axon guidance and signaling pathways such as *Egfr* (Figure 2f,g). Intriguingly, the subset of transcripts with increased m^6^A and also upregulated in Aβ42 brains included key stress genes such as *Hsp70Aa* (Figure 2g). Increased m^6^A levels on heat stress chaperones, however, results in a compromised stress response due to lower transcript stability and lower protein levels (Perlegos et al., 2022). Intriguingly, we found that knockdown of *Mettl3* in Aβ42 expressing brains worsens overall lifespan (Figure 2h), indicative of the importance of m^6^A regulation in the overall health of the disease brain. With m^6^A transcripts involved in critical neuronal signaling pathways downregulated with both age and disease, these data suggest that Aβ42 expression yields an overall premature aging signature on the brain with regard to m^6^A.

### 2.3 Knockdown of m^6^A methyltransferase *Mettl3* in neurons shortens lifespan

**FIGURE 3:**
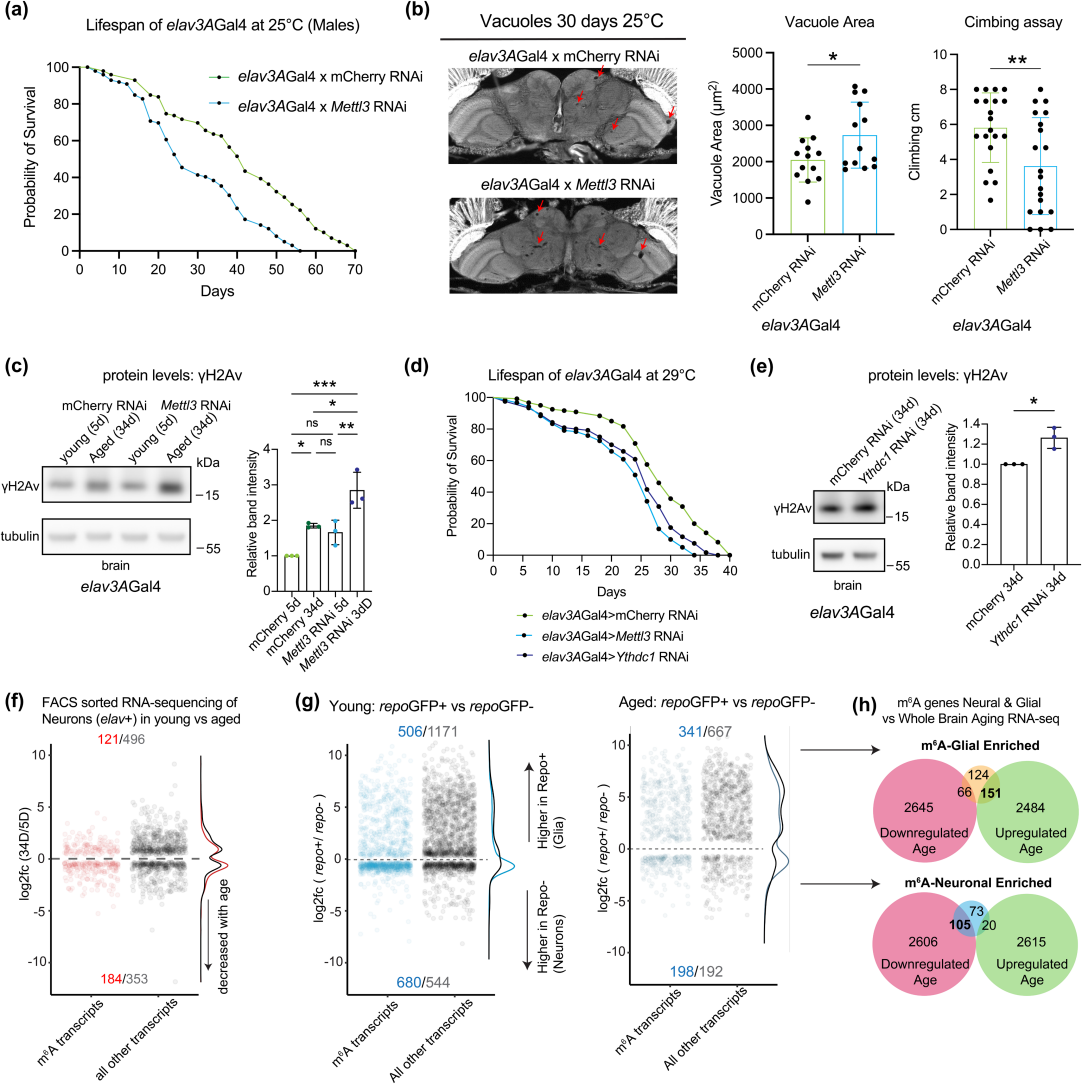
Reduction of m^6^A methyltransferase *Mettl3* in neurons decreased lifespan. (a) Lifespan of animals expressing control RNAi or *Mettl3* RNAi in neurons (*elav3A*Gal4 > mCherry RNAi vs. *elav3A*Gal4 > *Mettl3* RNAi). 25°C, *n* = 100, *n* = 100, ****p < 0.0001, Log‐rank test. (b) Paraffin sectioning of brains at 30d. Brain vacuoles highlighted by red arrows. Quantification of total vacuole area per brain calculated across 10 sections per brain, *n* = 12 brains. *p < 0.05, *t*‐test, *p* = 0.0337. Negative geotaxis assay to measure climbing ability at 30d. *n* = 20 flies. **p < 0.01, *t*‐test, *p* = 0.0064. (c) γH2Av levels in brain tissue, with age and *Mettl3* knockdown in neurons 5d versus 34d. (*elav3A*Gal4 > mCherry RNAi; *elav3A*Gal4 > *Mettl3* RNAi). *N* = 10 brains per replicate, 3 biological replicates. *p < 0.05, **p < 0.01, ***p < 0.001, ns = not significant, One‐way ANOVA, *p* = 0.0392, *p* = 0.0004, *p* = 0.0174, *p* = 0.0065. (d) Lifespan of animals expressing control RNAi, *Ythdc1* RNAi or *Mettl3* RNAi in neurons (29°C). *n* = 120, *n* = 120, *n* = 120 ****p < 0.0001, Log‐rank test. (e) Levels of γH2Av in brain tissue with *Ythdc1* knockdown in neurons 34d, *p < 0.05, *t*‐test, *p* = 0.0123. (f) Differentially expressed transcripts of *elav3A*GFP+ cells 34d versus 5d. Red are m^6^A transcripts, black all other transcripts. *p*_adj_ < 0.05. (g) Differential expression of *repo*GFP+ (Glial) cells versus *repo*GFP‐ (neuronal) cells at 5d (left) and 34d (right). Blue are m^6^A transcripts, black all other transcripts. Transcripts more highly expressed in *repo*GFP+ cells are considered glial enriched. (h) Comparison of glial‐enriched m^6^A transcripts (top) and neural‐enriched m^6^A transcripts (bottom) to the whole brain aging transcriptome 3d versus 50d (see Figure 1).

Based on the increased levels of m^6^A with age and disease, we extended these studies to investigate the functional consequences of reducing m^6^A on longevity and health span. Our m^6^A‐IP and RNA seq analysis consisted of whole brain or head tissue, and we sought to determine cell‐type specific effects of m^6^A in the brain in neurons versus glia. We found that knockdown of *Mettl3* selectively in neurons decreased the lifespan of animals (Figure 3a), as did neuronal knockdown of other reader and writer components in the m^6^A pathway (Figure S3a–e). Furthermore, neuronal *Mettl3* knockdown animals had increased vacuolization of the brain and decreased locomotor activity (Figure 3b), which are functional readouts of nervous system integrity. Additionally, we found that upregulation of *Mettl3* in neurons increased lifespan (Figure S3f,g). These data suggest that reducing levels of m^6^A in neurons is deleterious and promoting m^6^A is beneficial, indicating that m^6^A modification normally plays a critical role in maintaining neuronal health and animal longevity.

In mammalian cells, m^6^A RNA modification is essential for the DNA damage repair response by recruiting response factors to sites of damage (Xiang et al., 2017; Yu et al., 2021). Given the importance of DNA damage repair to postmitotic cells such as neurons with age, we examined levels of the DNA damage marker γH2Av (Jang et al., 2003). γH2Av levels normally increased with age in the brain, and *Mettl3* knockdown in neurons exacerbated the levels of γH2Av at both young and aged timepoints (Figure 3c). These results provide evidence to suggest that one role of *Mettl3* in the brain may be for protection against DNA damage as neurons age. Recent studies have also implicated nuclear reader protein Ythdc1 in the DNA damage response (Widagdo et al., 2022; Zhang, Chen, et al., 2020), to stabilize DNA–RNA hybrids and recruit damage machinery. We found that *Ythdc1* RNAi in the *Drosophila* brain was also associated with decreased lifespan of animals (Figure 3d) and increased levels γH2Av at 34d (Figure 3e). *Ythdc1* may also play a role in DNA damage recovery in the *Drosophila* brain with age.

To gain greater insight into the cellular regulation of m^6^A genes in neurons, we separated neurons from glia to define the dynamics of m^6^A marked transcripts with age in neurons. FACS sorting of GFP tagged *Drosophila* brain cells allowed enrichment of neurons (*elav*GFP+) versus glia (*repo*GFP+), which we performed with and without *Mettl3* knockdown. Similar to whole brain analysis, m^6^A marked transcripts were more downregulated in *elav*GFP+ cells with age (Figure 3f). PCA analysis highlighted that most of the variability in the *elav*GFP+ samples was associated with PC1 which corresponded to age, while PC2 was largely driven by transcriptional changes of *Mettl3* knockdown (Figure S3e). The *Mettl3* RNAi samples were shifted along the PC1 axis, indicating that they appear more aged transcriptionally than the control samples (Figure 3e). Differentially expressed transcripts that may contribute to these changes in *Mettl3* RNAi *elav*GFP+ cells include those for toll signaling and fatty acid metabolism pathways (Figures S3f and S4a,b). Abnormal regulation of these metabolic signaling pathways may be responsible for some of the deficits observed in neurons upon *Mettl3* knockdown.

### 2.4 The transcriptional response of m^6^A genes in glia with age

Glial cells are also crucial players to brain health and aging (Freeman, 2015), and undergo significant changes in response to injury and inflammation, releasing molecules that can lead to neuronal damage and degeneration (Hickman et al., 2018; Zuchero & Barres, 2015). Previously, we found that there is robust glial activation to traumatic brain injury (TBI) in the *Drosophila* brain that mimics the upregulated transcriptional response that occurs with normal brain aging (Byrns et al., 2021). Because of these data, we examined the subset of genes upregulated with both age and TBI: we found that many of these transcripts, including *Mmp1*, *puc*, *Ets21C*, and *dorsal*, are m^6^A marked and have increased m^6^A levels with age (Figure S5a,b). These transcripts were also distinct in that they are more highly expressed in glial cells versus neurons (Data S4).

To further compare the transcriptional response of neurons versus glia with age, equal numbers *repo*GFP+ (glial) and *repo*GFP− (neural) cells were FACS sorted from brains at 5d versus 34d. Analysis showed that most m^6^A transcripts in young animals were not differentially expressed between these cell types (*repo*+ 506 genes and *repo*− 680 genes), with a slight trend toward higher expression of m^6^A transcripts in neuronal cells (Figure 3g). With age, however, there was a density shift with more m^6^A transcripts being highly expressed in *repo*+ (341 genes) versus *repo*− (198 genes) cells (Figure 3g). Moreover, neuronal enriched m^6^A transcripts (*repo*GFP− population) shifted toward downregulation with age (Figure 3h). By contrast, *repo*GFP+ enriched m^6^A transcripts were shifted toward upregulation with age (Figure 3h), including *Mmp1, puc, Ets21C*, and *dorsal* which are upregulated with age (see Figure S5a). Overall, a unique subset of transcripts that have increased m^6^A levels and increased expression with age are more highly expressed in glia. These data indicate neurons versus glia have at least partially divergent avenues of m^6^A gene regulation with age.

### 2.5 The functional role of *Mettl3* in glia with age and disease

**FIGURE 4:**
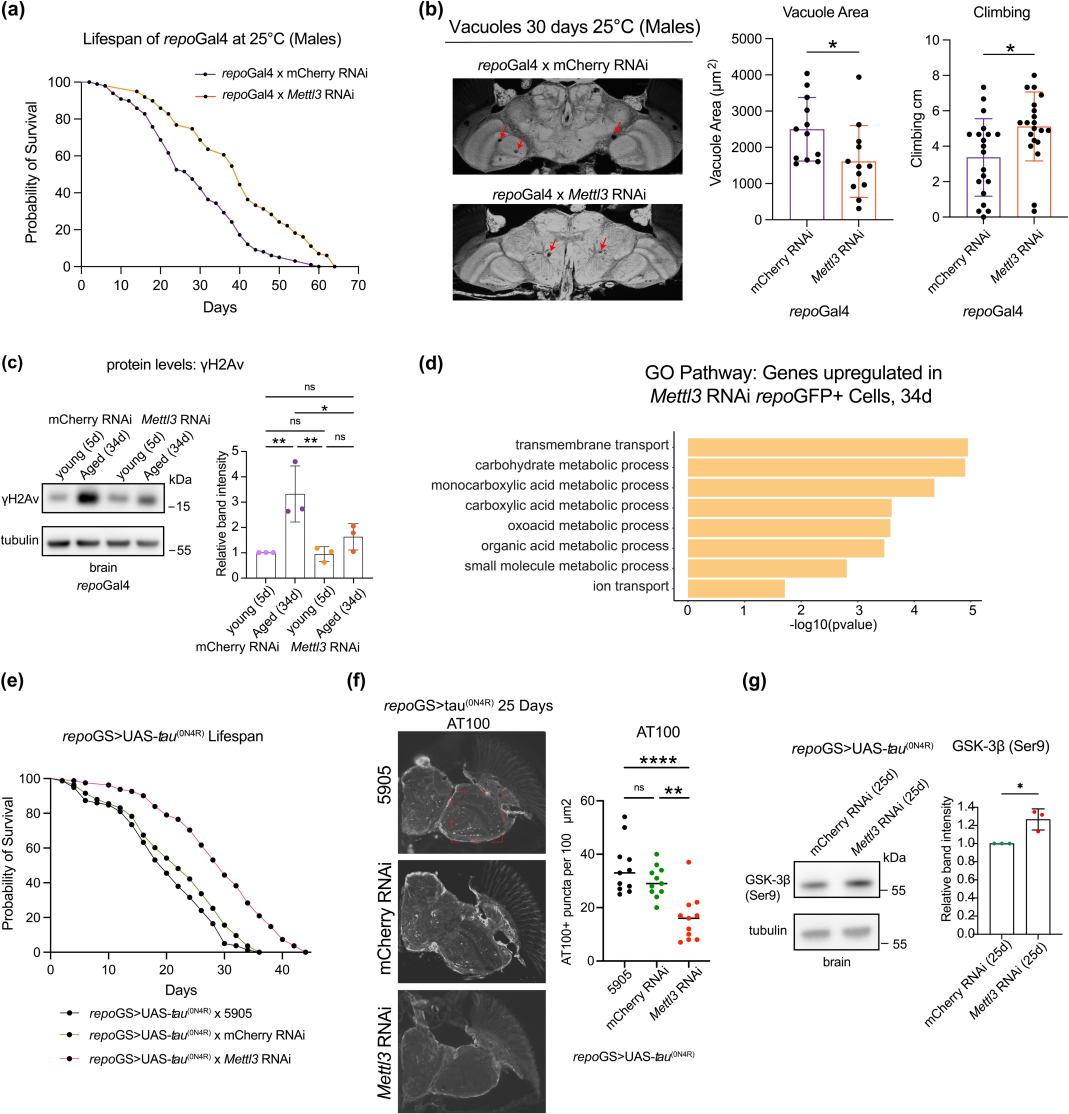
Reduction of *Mettl3* in glia increased lifespan. (a) Lifespan curve of animals expressing control RNAi or *Mettl3* RNAi in glia. (*repo*Gal4 > mCherry RNAi versus *repo*Gal4 > *Mettl3* RNAi). *n* = 100, *n* = 100, ****p < 0.0001, Log‐rank test. (b) Paraffin sectioning of brains at 30d. Brain vacuoles highlighted with red arrows. Quantification of total vacuole area per brain calculated across 10 sections per brain. *n* = 12 brains, *p < 0.05, *t*‐test, *p* = 0.0295. Negative geotaxis assay to measure climbing ability for each genotype at 30d. *n* = 20 flies, *p < 0.05, *t*‐test, *p* = 0.011. (c) Levels of γH2Av in brain tissue with age and *Mettl3* knockdown in glial cells (5d vs. 34d). (*repo*Gal4 > mCherry RNAi; *repo*Gal4 > *Mettl3* RNAi). *n* = 10 brains per replicate, 3 biological replicates. *p < 0.05, **p < 0.01, ns = not significant, One‐way ANOVA, *p* = 0.0085, *p* = 0.0073, *p* = 0.0436. (d) GO analysis of upregulated genes in *Mettl3* RNAi *repo*GFP+ cells versus mCherry RNAi cells at 34d. Differentially expressed transcripts of *repo*GFP+ cells *p*_adj_ < 0.05. (*repo*GFP+ > mCherry RNAi versus *repo*GFP+ > *Mettl3* RNAi). (e) Lifespan of animals expressing human wild type tau (0N4R) in glial cells *repo*GS > UAS‐tau^(0N4R)^ with control (BL5905), mCherry RNAi, or *Mettl3* RNAi. Lifespan carried out at 29°C. *n* = 80, *n* = 83, *n* = 81, ****p < 0.0001, Log‐rank test. No significance (ns) between BL5905 and mCherry RNAi *p* = 0.0778. (f) Paraffin immunostaining of AT100 tau phosphorylation in brains, 25d. Quantification of puncta from the same 100 mm^2^ region of all brain sections, *n* = 11 brains per genotype, ****p < 0.0001, **p < 0.01, ns = not significant, One‐way ANOVA, *p* < 0.0001, *p* = 0.0013. (g) Levels of GSK‐3β (Ser9) phosphorylation with *repo*GS > UAS‐tau^(0N4R)^ brains in control versus *Mettl3* knockdown, *n* = 10 brains per replicate, 3 biological replicates, *p < 0.05, *t*‐test, *p* = 0.0166.

Given these striking differences in glia versus neurons, we assessed the functional role of m^6^A in glia. Surprisingly, in contrast to knockdown in neurons, *Mettl3* knockdown in glia using the glial specific driver *repo*‐GAL4 led to extended animal lifespan, improved locomotor activity and decreased brain vacuolization with age (Figure 4a,b and Figure S5c–g). Upregulation of *Mettl3* in glial cells had the opposite effect and modestly reduced overall longevity (Figure S5h). Moreover, the DNA damage response of the brain with *Mettl3* knockdown in glia was reduced, with lower levels of γH2Av, indicating less genotoxic stress (Figure 4c). We further analyzed FACS sorted *repo* + cells with and without *Mettl3* RNAi at 5d versus 34d. PCA analysis of the overall transcriptional effects showed that PC1 corresponded with age, and the 34d older *Mettl3* RNAi glia were shifted toward the younger 5d glial cells, indicating an overall less aged profile (Figure S5i). Differentially expressed transcripts in *Mettl3* RNAi *repo*GFP+ cells (34d) highlighted an enrichment for small molecule and carboxylic acid metabolic processing pathways (Figure 4d), indicating alterations in these metabolism pathways may shift *Mettl3* RNAi cells toward a younger profile. Overall, these data indicated that whereas *Mettl3* function in neurons is normally beneficial with age, *Mettl3* function in glia is deleterious. Given the prominent role of glia in brain integrity, and that many genes upregulated with age are glial enriched and m^6^A modulated, these data indicate that *Mettl3* gene activity in glia may be a strong contributor to integrity of the brain with age.

### 2.6 *Mettl3* knockdown extends lifespan in a glial tauopathy model

To gain insight into the impact of m^6^A and disease toxicity in glial cells, we examined the impact of *Mettl3* in a model of glial tauopathy. Abnormal phosphorylation and accumulation of the microtubule‐associated protein tau occurs with age and disease; whereas some diseases including Alzheimer's have enriched pathological tau largely in neurons, other diseases like progressive supranuclear palsy, corticobasal degeneration, and TBI show pathological tau accumulation in both neurons and glia (Berry et al., 2001; Schmidt et al., 2001; Tagge et al., 2018). Expression of human tau in glial cells models glial tauopathy, with reduced lifespan, increased pathological tau accumulation, and brain vacuolization (Byrns et al., 2021; Colodner & Feany, 2010). *Mettl3* knockdown in glia, however, significantly reduced AT100 pathological tau puncta and extended animal lifespan (Figure 4e,f). There were no changes in overall Tau protein level (Figure S5j). *Mettl3* knockdown in neurons expressing tau had only a slightly increased lifespan (Figure S5k). Glycogen synthase kinase 3 beta (GSK3β) is a protein kinase that phosphorylates tau, including at the AT100 pathological site. Glial knockdown of *Mettl3* increased ser9 phosphorylation levels of GSK3β protein (Figure 4g), which is predicted to decrease its activity and ability to phosphorylate tau. These data underscore that *Mettl3* knockdown in glia improves animal health span normally and also upon tau toxicity.

### 2.7 TRAP profiling from neurons versus glial cells uncovers cell‐specific m^6^A regulation of translation

**FIGURE 5:**
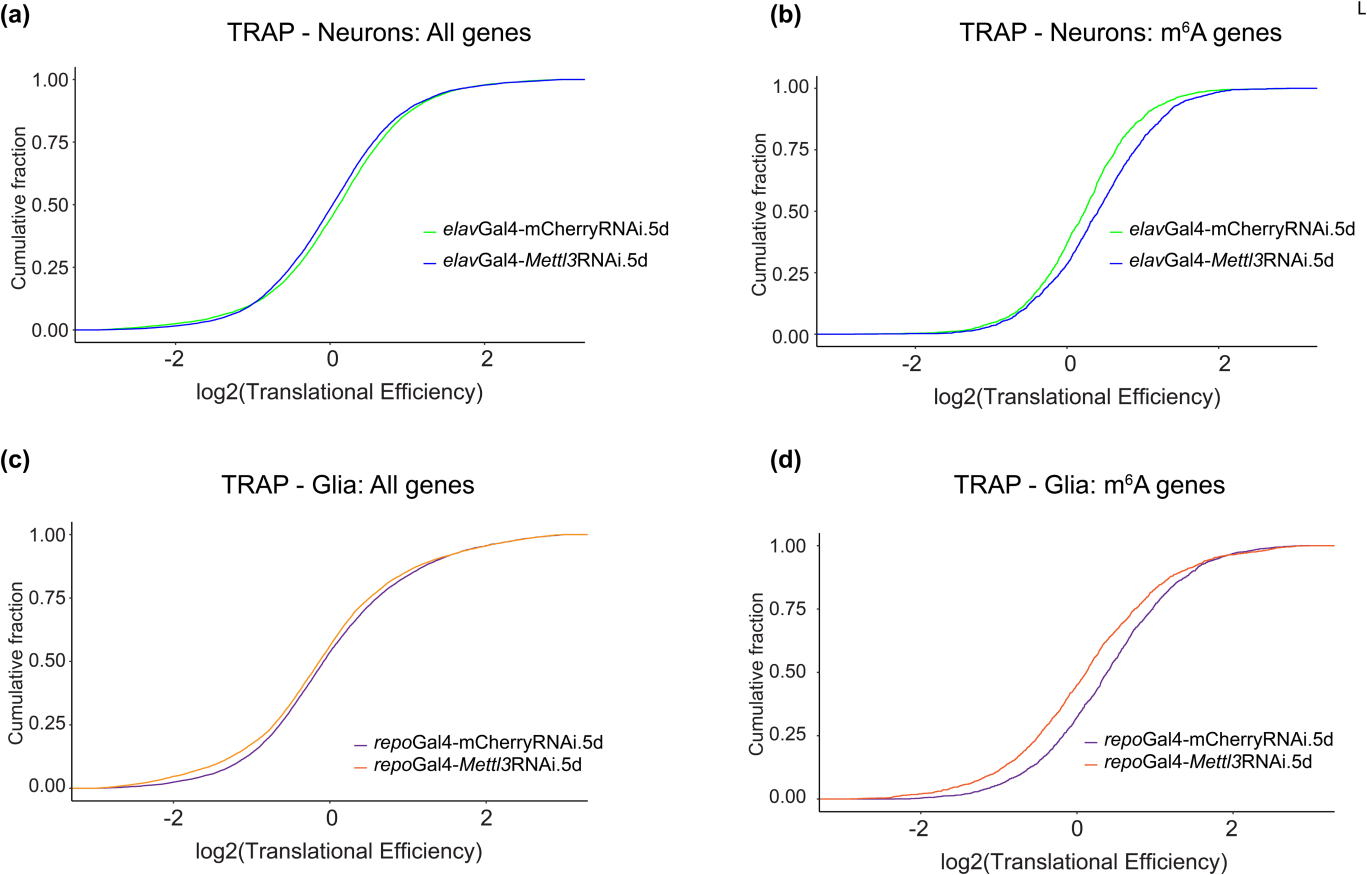
Translational profiling from neurons and glia with age and *Mettl3* RNAi. Translational efficiency determined from TRAP assay with *Mettl3* knockdown in (a, b) neurons. (*elav3A*Gal4 > UAS‐RpL3‐FLAG × mCherry RNAi vs. *Mettl3* RNAi) and (c, d) glia (*repo*Gal4 > UAS‐RpL3‐FLAG × mCherry RNAi vs. *Mettl3* RNAi) for in all expressed genes versus m^6^A modified transcripts.

These findings suggested that the function of m^6^A in glia versus neurons is markedly distinct: in neurons, m^6^A normally protects the brain with age; by contrast, in glia, m^6^A tags transcripts that contribute to age‐associated decline. m^6^A modification can impact many aspects of transcript dynamics, including stability which is reflected in the transcript levels, but also the translation of transcripts. Given the divergent roles of m^6^A in neurons versus glia, we determined if m^6^A knockdown in these two cell types differentially impacted translational efficiency. We used translating ribosome affinity purification (TRAP) with FLAG‐tagged RPL3 to isolate and sequence the population of mRNAs that are associated with the 80S ribosome in neurons versus glia, normally and upon *Mettl3* knockdown. Analysis of neurons indicated an increased translational efficiency of m^6^A‐tagged transcripts upon *Mettl3*‐knockdown versus all transcripts, which showed no change in ribosome association (Figure 5a,b). This indicates that, normally, m^6^A‐marked transcripts in neurons have lower translational efficiency. By contrast, *Mettl3* knockdown in glia showed decreased ribosome association of m^6^A transcripts (Figure 5c,d). This indicates that, normally, m^6^A marked transcripts show higher translational efficiency in glial cells. Taken together, these data indicate that m^6^A functionally limits translation efficiency in neurons, but by contrast promotes translational efficiency in glia.

## 3 DISCUSSION

**FIGURE 6:**
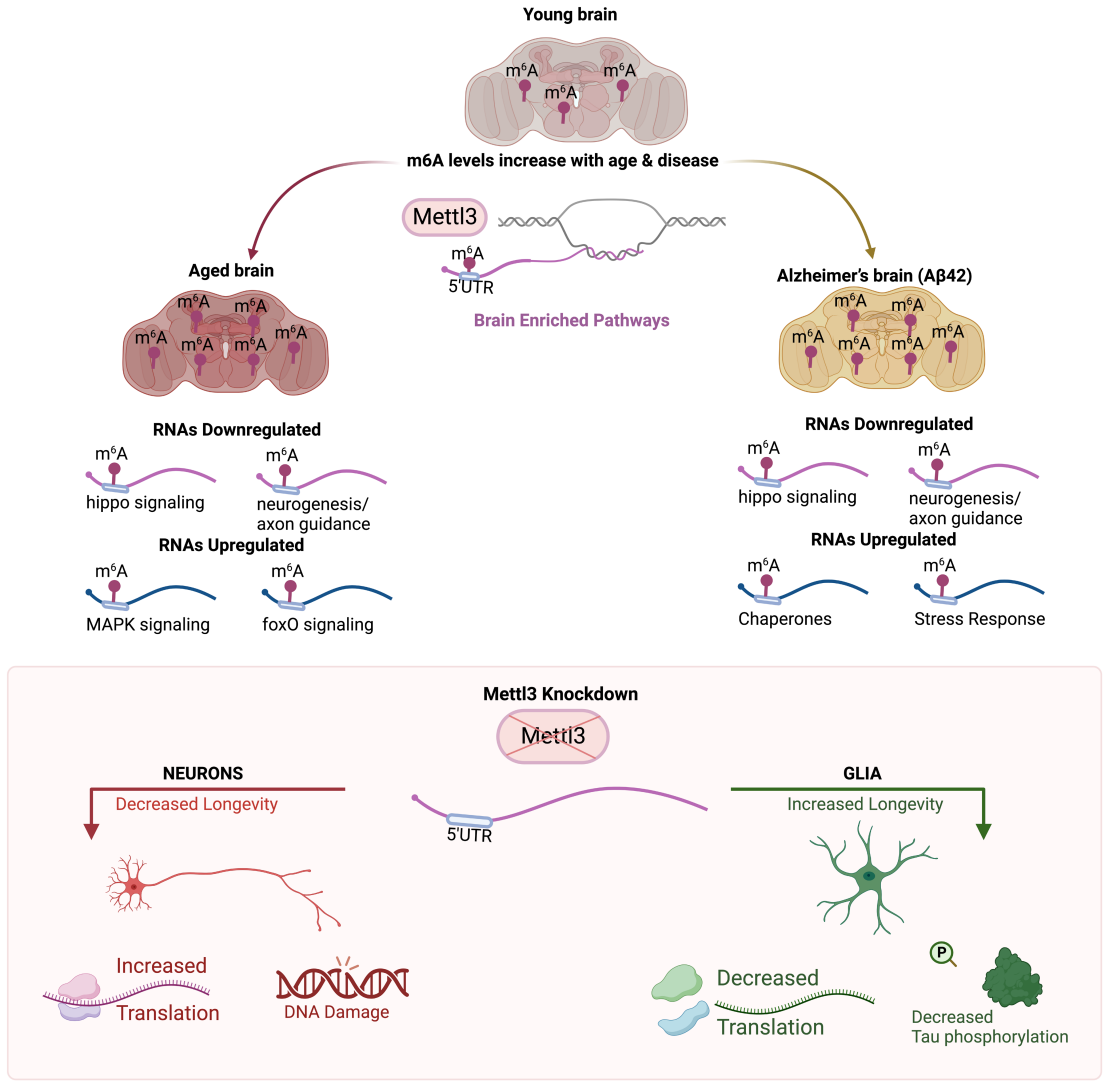
m^6^A regulation in the brain with age and disease. Model of m^6^A regulation in the brain with aging. Top: m^6^A levels increase with age and in the brains of animals expressing human Aβ42 in neurons. m^6^A transcripts were mostly downregulated with age and with disease, and are enriched for neurogenesis and signaling pathways. Bottom: Knockdown of *Mettl3* in neurons decreases lifespan and health span, increases translation efficiency of m^6^A transcripts, and increases DNA damage. These data suggest *Mettl3* function is normally protective to neurons. Knockdown of *Mettl3* in glia promotes lifespan and health span, and decreases translation efficiency of m^6^A modified transcripts. *Mettl3* knockdown in glial cells also extends lifespan of animals expressing human tau, and mitigates tau phosphorylation pathology. These data indicate that *Mettl3* activity is normally deleterious to glial function.

m^6^A is highly prevalent in the brain and impacts various aspects of RNA metabolism, including mRNA stability and translation. Here we provide insight into the role of m^6^A in the adult *Drosophila* brain in vivo with age and disease, and in neurons versus glia. An initial finding is that m^6^A modification increases in the 5' UTR of transcripts in the brain with both age and disease. This increase has a negative effect on the mRNA expression levels of the tagged transcripts such that these transcripts become, on average, more downregulated. However, when examining m^6^A in neurons versus glia, we find that there are different functional outcomes of *Mettl3* knockdown in the two cell types. Decreasing *Mettl3* in neurons decreases animal longevity and functional readouts, indicating that *Mettl3* function normally promotes neural health. By contrast, decreasing *Mettl3* in glial cells conferred resilience to functional decline and extended lifespan, indicating that *Mettl3* normally limits glial cell function. The two cell types also regulated translation of m^6^A modified RNAs in an opposite manner—*Mettl3* normally functions to decrease translation efficiency of m^6^A tagged transcripts in neurons, whereas *Mettl3* promotes translation efficiency of tagged transcripts in glia. The different translational efficiencies presumably reflect differences in the levels or types of m^6^A reader proteins expressed in neurons versus glia which confer these different biological impacts. Taken together, these data highlight a critical and opposite functional impact of m^6^A modified transcripts in neurons versus glia in brain integrity and health span with age and disease (Figure 6).

### 3.1 m^6^A modification in the brain with age and chronic stress

The aging brain is highly susceptible to stress, and hallmarks of aging include increased proteotoxic protein accumulation and decreased RNA transcription (Gemma et al., 2007; Landis et al., 2012; Leak, 2014). Age is also a major risk factor for cognitive decline and degenerative disease. As the brain ages, cellular pathways required for the key functions of neurons become downregulated, while stress pathways that deal with protein misfolding, DNA damage, and ROS become upregulated (Ham & Lee, 2020; Sen et al., 2016). Although different pathways and genes that change with age differ depending on the cell type, aging is associated with chronic stress activation.

Previously, we found that m^6^A increases on polyA+ RNA with acute stress, but that loss of *Mettl3* and nuclear reader *Ythdc1* confer resilience to acute stress. m^6^A‐IP sequencing showed that only ~7 out of 98 known heat stress chaperones in *Drosophila* are m^6^A marked, but these include critical chaperones like the *Hsp70s* at basal conditions (leading to their upregulation, and presumably providing stress preconditioning protection upon *Mettl3* knockdown). Rather, m^6^A transcripts in the brain comprise a select subset (~14%) of brain‐expressed genes that are enriched for neurogenesis and signaling pathways such as MAPK, HIPPO, foxO, and TGF‐beta signaling—all critical signaling pathways for dynamic functioning of the brain. Thus, m^6^A plays a critical role in fine tuning functional signaling pathways. Our data here show that m^6^A levels increased in the 5' UTR on many transcripts with age; these genes remain enriched for functional signaling pathways. m^6^A is known to mark transcripts for degradation (Du et al., 2016; Fu & Zhuang, 2020; Li et al., 2019; Shi et al., 2017), and increased m^6^A in the 5' UTR with age is negatively correlated with RNA expression level. Thus, one mechanism by which key genes in dynamic signaling pathways may become downregulated functionally with age in the brain may be that they are m^6^A modified.

In mammalian cells, m^6^A is associated with the DNA damage response, with damage recruiting METTL3 and causing it to bind DNA lesions and promote m^6^A modification of RNAs from damaged chromatin (Zhang, Chen, et al., 2020). Ythdc1 recognizes the m^6^A modified RNAs and recruits proteins to promote homologous recombination (HR)‐mediated repair (Widagdo et al., 2022; Xiang et al., 2017; Zhang, Chen, et al., 2020). In the fly brain, we found that the marker of DNA damage, phosphorylated H2Av (γH2Av) (Grigorian et al., 2017), is increased with age and that *Mettl3 or Ythdc1* reduction in neurons further increased γH2Av. This association suggests an m^6^A triggered pathway combating DNA damage occurs in neurons in the fly brain and may contribute to the decreased survival and increased brain degeneration upon loss of *Mettl3*. By contrast, *Mettl3* knockdown in glial cells conferred the opposite response of decreased levels of γH2Av. The response in fly neurons is consistent with the DNA repair outcome observed in mammalian cells, whereas the response in glia is distinct.

We observed altered levels of m^6^A machinery proteins in the brain with age, which may explain the overall increased levels of m^6^A with age. The cytoplasmic reader protein Ythdf is known in *Drosophila* to be crucial for learning and memory (Kan et al., 2021), and in mammalian cells for shuttling 365 m^6^A‐marked transcripts into P‐bodies and stress granules for RNA decay (Du et al., 2016; Fu & Zhuang, 2020). Because reducing the expression of Ythdf in neurons had a negative impact on lifespan (see Figure S3c), a decline in Ythdf levels during aging may have an unfavorable effect on overall health. 368 Nuclear reader protein Ythdc1 has recently been shown in mammalian cells to lead to decay of 369 chromatin binding RNAs and lncRNA transcripts (Ji et al., 2021; Liu, Dou, et al., 2020), as well as recruiting DNA damage 370 machinery and assisting in repair mechanisms (Xiang et al., 2017; Yu et al., 2021; Zhang, Chen, et al., 2020). Here we find Ythdc1 protein levels in the 371 *Drosophila* brain normally increase with age and *Ythdc1* knockdown in neurons decreased lifespan—if Ythdc1 functions in the DNA damage response in *Drosophila* neurons, the increase with age would be protective to the brain.

### 3.2 m^6^A modification and brain degeneration

m^6^A regulation with expression of Alzheimer's Aβ42 was similar to normal aging in that m^6^A levels increased in the 5' UTR of transcripts enriched in synaptic functionality and dynamic signaling. The m^6^A genes contrast with all transcripts downregulated with Aβ42 or age, the latter 379 of which are largely enriched in oxidative phosphorylation and TCA cycle. Aβ42 brains, however, also showed a unique response in that transcripts upregulated were enriched for stress and chaperones pathways. Thus, the proteotoxic stress of human Aβ42 expression activates stress chaperone pathways. Notably, among those genes with increased 5' UTR m^6^A were *Hsp70* transcripts. Previously, we showed that *Hsp70* is tagged by m^6^A basally, but not with acute stress, and that m^6^A serves to reduce transcript and protein levels; Hsp70 protein is increased and the animals stress resilient with *Mettl3* knockdown (Perlegos et al., 2022). Taken together, these findings indicate that the increased m^6^A on *Hsp70* transcripts may especially compromise the stress response of Aβ42 expressing animals. Studies in mice show that m^6^A levels are elevated in the cortex and the hippocampus of APP/PS1 transgenic mice compared to controls, with Mettl3 upregulated, and the demethylase FTO downregulated (Han et al., 2020). Similar changes in m^6^A machinery occur in the *Drosophila* brain, with increased levels of Mettl3 protein in Aβ42 brains. Since *Mettl3* loss in neurons is normally deleterious with age, these data suggest that the increase in m^6^A in Aβ42 expressing animals may be part of a protective response of the brain against this toxic insult. Consistent with *Mettl3* upregulation being protective, knockdown of *Mettl3* in Aβ42 expressing neurons reduced lifespan even further (see Figure 2h).

Unexpectedly, *Mettl3* knockdown in glia functioned opposite to knockdown in neurons, by conferring increased longevity normally and in glial tauopathy. m^6^A is a critical regulator in glioblastoma and loss of METTL3 is beneficial to radiosensitivity of disease cells (Cui et al., 2017; Dong & Cui, 2020; Li et al., 2019; Zhang et al., 2017). m^6^A also stabilizes and increases the translation of proto‐oncogenes in glioblastoma (Cui et al., 2017; Dong & Cui, 2020; Li et al., 2019; Zhang et al., 2017). Although the majority of 5' UTR modified transcripts were decreased with age, transcripts in select signaling pathways including MAPK and foxO signaling (See Figure S1g) were upregulated with age; these transcripts were enriched in *repo*GFP+ glial cells. Thus, m^6^A is poised to be a significant regulator of glial‐associated disorders, functioning in a manner opposite from neurons. Overall, m^6^A regulation seems dependent on the targets affected and the cell types examined and brain tissue shows high m^6^A modification, with a distinct methylome. (Dominissini et al., 2012; Liu, Li, et al., 2020).

### 3.3 Translation regulation

*Mettl3* manipulation in the fly brain has a very different translational impact (Perlegos et al., 2022) from that of *Drosophila* epithelial S2 cells (Kan et al., 2021) and some situations of human cultured cells (Meyer et al., 2015). Here, we extended these observations by immunoprecipitating ribosome associated RNAs from neurons versus glia, with or without *Mettl3* knockdown. In neural cells, *Mettl3* loss led to increased translational efficiency, while in glial cells it decreased translational efficiency. These findings indicate that each cell type, with its distinct expression repertoire of genes (presumably including central m^6^A reader proteins), has an enormous impact on the functional outcome of m^6^A‐modified transcripts. Moreover, a subgroup of transcripts show increased m^6^A with age, are upregulated with age, and are more highly expressed in glia versus neurons. m^6^A transcripts are also normally more efficiently translated in glia. These transcripts have been shown to be critical to the overall response of the brain to TBI and to normal aging, and chronic activation of these genes in glia is detrimental (Byrns et al., 2021). Thus, these transcripts may be key genes that confer the increased health span with *Mettl3* knockdown in glia (see Figure S5a,b). Given that some of these genes function in glia to extend lifespan (Bolukbasi et al., 2021), these findings suggest a nuanced regulation of key transcripts and m^6^A modification. These datasets set the stage for future studies defining additional nuances of m^6^A transcripts and their regulation in different subtypes of neurons (as part of different circuits, expressing different neurotransmitters, for example), and glia (cortical involved in the blood–brain barrier, versus ensheathing, for example).

### 3.4 Concluding remarks

Our data indicate that m^6^A transcripts, which are enriched in critical signaling and neuronal pathways, are regulated during age and disease, and differentially regulated in neurons versus glia. Whereas *Mettl3* function in *Drosophila* is protective to neurons, it is deleterious to glia, and these differential responses may have a large impact on the integrity of the brain with age and with disease. Given that GWAS hits for Alzheimer's disease are enriched in genes expressed in neurons, but also glia (Bellenguez et al., 2022), the distinct impact of m^6^A regulation of transcripts in neurons versus glia is poised for an important contribution to the maintenance and integrity of the brain with age and its response to disease.

## 4 MATERIALS AND METHODS

### 4.1 *Drosophila* stocks and lifespan analysis

A full list of *Drosophila* stocks used in this study are in Data S1. RNAi and UAS lines were generated by the Harvard Transgenic RNAi Project (TRiP) (Johnson et al., 2020), and stocks were obtained from the Bloomington *Drosophila* stock center, Indiana, USA. Crosses were performed at 25°C and grown on standard cornmeal molasses agar. Driver lines used as indicated per experiment, *elav*Gal4‐3A, *elav* ^C155^, *elav*GS, *repo*Gal4, *repo*GS. For all experiments male flies were used, for consistency in the experiments, and to avoid issues in food due to egg laying of females. All experiments were performed at 25°C unless otherwise indicated. For lifespan analysis flies were flipped to fresh vials every 2d and housed at 25°C or 29°C (for accelerated lifespan analysis) on a 12 h light/dark cycle. For GeneSwitch (inducible gal4‐UAS) experiments, food was prepared with either 100 μL of RU486 (4 mg/mL in 100% EtOH; Sigma‐Aldrich, #M8046–1G) pipetted onto food vials and allowed to dry for 24 h. Flies were put onto RU486 food as adults 1–2 days post eclosion. The number of dead flies was recorded after each time flipping, every 1–2 days.

### 4.2 Brain dissections

Brain dissections were conducted as previously described (Kennerdell et al., 2018). Briefly, flies were anesthetized using CO_2_ and decapitated using forceps. The head was placed posterior side down and the proboscis was then removed using Dumont #5S forceps (Fine Science Tools, #11254‐20). The brain was then gently popped out through the proboscis cavity, cleaned in PBS, and transferred to an RNAse free microfuge tube and PBS was aspirated. Brains were then ground in Laemmli Buffer (5 μL per brain, at least 10–20 brains for each sample) for western immunoblotting, or Trizol for RNA analysis.

### 4.3 Western immunoblot analysis

Brain or head samples were homogenized in sample buffer of 1x Laemmli sample Buffer (BioRad, 1610737), 50 μL β‐mercaptoethanol (Sigma, #M6250), 1x protease inhibitor (Roche, #11836170001), and 1 mM PMSF (Sigma, #P7626). 5 μL of sample buffer was added per brain, 7.5 μL added per head, and 40 μL added per whole fly. Samples are boiled at 98°C for 3 min, and then centrifuged at 1500 rpm for 3 min at room temp. Sample was loaded onto 15 well 1.0 mm 4%–12% Bis‐Tris NuPAGE gels (Thermo Fisher, WG1401) with pre‐stained protein ladder (Thermo Scientific, #22619). 1 brain or head equivalent was loaded on each lane per experiment. Gel electrophoresis was performed using Xcell Surelock Mini‐Cell Electrophoresis System at 140 V, and transferred overnight onto a nitrocellulose membrane 0.45 μM (Bio‐rad, #1620115), using a Bio‐rad mini transblot cell at 90A for 16 h. Membranes were stained in Ponceau S (Sigma, #P71701L), washed in double distilled water, and imaged with Amersham Imager 600. Ponceau S was washed off in 3 × 5 min in Tris‐buffered saline with 0.1% Tween20 (TBST). Membrane was blocked in 5% nonfat dry milk (LabScientific, #M08410) in TBST for 1 h, and incubated with primary antibodies with blocking buffer overnight at 4°C. Following 3 × 5 min washed in TBST, membranes were incubated with HRP‐conjugated secondary antibodies at 1:5000 for 1 h at room temp in blocking solution. Membranes were washed 3 × 5 min in TBST and the signal was developed using ECL prime (Cytivia, #RPN2232) and detected using an Amersham Imager 600. Primary antibodies used: anti‐tubulin (1:5000, DSHB, #AA4.3, Lot.5/31/18‐44ug/ml), anti‐Mettl3 (1:5000, Proteintech, #15073‐1‐AP, Lot.Ag7110), anti‐tau (1:100,000, DAKO, #A0024, Lot.20024929), anti‐yH2Av (1:500, DSHB, #UNC93‐5.2.1), anti‐GSK‐3β(ser9) (1:3,000, Cell Signaling Technology, #9336). Rabbit anti‐Ythdc1 (1:5000) affinity purified rabbit antibodies was generated by Biosynth against 18 residues of Ythdc1 (157‐173 “CRTKIPSNANDSAGHKSD”) as described (Perlegos et al., 2022). Secondary Antibodies used: Goat anti‐mouse (1:5000, Jackson lmmunoResearch, #115‐035‐146, Lot.153978), Goat anti‐Rabbit (1:5000, Jackson lmmunoResearch, #111‐035‐144, Lot.138306), Goat anti‐rat (1:5000, Thermo Fisher Scientific, #A10549, Lot.2273679).

### 4.4 Paraffin sectioning

Fly heads were decapitated and fixed in Bouin's solution (Sigma‐Aldrich, #HT10132) for 6d. Fixation was stopped by submersion in leaching buffer (50 mM Tris pH 8.0, 150 mM NaCl) overnight at RT. Heads were then processed through graded EtOH dehydration at the following times and concentrations: 30 min 70%, 30 min 90%, 30 min 95%, 30 min 100%, 30 min 100%, followed by xylenes (2x, 30 min) and finally fixed in paraffin (2 h). Heads were blocked and sectioned into 8 μM thick ribbons placed onto glass slides. Ribbons were deparaffinized by heating at 65°C for 1 h followed by washes in histoclear (VWR; #101412–876) for 5 min. For assessing brain vacuolization, sections were mounted with Cytoseal XYL (ThermoFisher, #8312–4). Sections were imaged on a Leica DFC360 FX under 10x objective, 1.2x magnification with fixed exposure settings. Fly brain tissue is auto‐fluorescent for pigmented eyes thus signal was detected using an I3 filter cube. Brain vacuolization was quantified in FIJI. For each brain, vacuolized area were averaged across 10 nonconsecutive sections to determine average brain vacuolization. Image acquisition and analyses were performed blind to sample identity.

### 4.5 Immunofluorescence on paraffin sections

Paraffin sections of fly brains expressing human tau (0N4R) in glia were rehydrated to water as follows: 100% EtOH for 3 min, 100% EtOH for 3 min, 95% EtOH for 1 min, 80% EtOH for 1 min, H_2_O for 5 min. Antigen retrieval was performed by boiling slides in citric acid buffer (Vector; #H‐3300) at 95°C for 40 min. Sections were then permeabilized in PBS with 0.1% Tween‐20 (PBST), then blocked in 3% bovine serum albumin (BSA) for 1 h at RT, then incubated in 1° antibody (AT100, Invitrogen, #MN1020, Lot.UH2805521) 1:200 overnight at 4°C. Sections were washed (2x, PBST) then incubated in 2° antibody for 1 h at RT. Sections were washed in PBST, rinsed in deionized water then cover slipped with mounting media. For phospho‐tau puncta quantification, sections were imaged on a Leica DFC360 FX under a 10x objective, 1.2x magnification and identical exposure settings. Puncta were counted in FIJI using the cell counter. Puncta were counted in one brain hemisphere per 100 *μ*M (Haigis & Yankner, 2010). Image acquisition and analysis was performed blind to sample identity.

### 4.6 Climbing assay

Individual flies were placed in empty vials and allowed to acclimate for 30 min. Climbing was measured by gently tapping flies to the bottom of the vial then recording height climbed after 30 s. Climbing height for each animal was assessed in FIJI and averaged across three trials taken within a 10 min testing interval. Max vial height of 8 cm.

### 4.7 RNA extraction

Tissue was homogenized in 200 μL of Trizol (Thermofischer Scientific, #15596026) in RNase free 1.5 mL microfuge tubes (Thermofischer scientific, #AM12400). 800 μL of Trizol (Thermofischer scientific, #15596026) was added per tube and 200 μL of chloroform (Fisher Scientific, #AC423555000) was added and tubes vigorously shook for 20 s at room temperature. Samples were left for 5 min at RT to form upper aqueous phase, and centrifuged at 4°C for 15 min at 12,000 × ***g***. The upper aqueous phase was transferred to a fresh RNase free tube. RNA samples were then processed using the Zymo RNA clean & concentrator‐5 kit (Zymo, #R1013), using their RNA cleanup from aqueous phase after Trizol /chloroform extraction protocol plus on column with DNaseI treatment. RNA amount was measured using a nanodrop and integrity was validated through on an Agilent 2100 Bioanalyzer using an RNA nano chip.

### 4.8 m^6^A‐IP sequencing

Total RNA was extracted from 200 *Drosophila* heads per replicate using Trizol/ chloroform extraction. PolyA+ mRNA was obtained using NEBNext Poly(A) mRNA Magnetic Isolation Module. PolyA+ RNA was fragmented using the NEB Next Magnesium Fragmentation Module (NEB, E6150S) for 4 min at 95°C for a 250 ng sample of polyA+ RNA, and RNA was repurified using the Zymo RNA clean & concentrator‐5 kit (Zymo, #R1013). Ten percent of the fragmented polyA+ RNA was saved as an input control for sequencing. M^6^A‐immunoprecipitation was done using the EpiMark N6‐Methyladenosine Enrichment kit protocol with some minor alterations. 30 μL of protein.

G magnetic beads (NEB, #S1430) were washed and resuspended in IP buffer (150 mM NaCl, 10 mM Tris‐HCL, 0.1% NP‐40). 4 μL of synaptic systems antibody (Synaptic systems, #202003, Lot.2116) was conjugated to protein G‐magnetic beads (NEB, #E1611A) for 2 h at 4°C. Beads/antibody were washed twice in IP buffer. ~1 μg PolyA+ RNA was incubated with beads/antibody in IP buffer supplemented with 0.1% SUPERase‐In RNase Inhibitor (Thermo Fisher; #AM2696) for 2 h at 4°C. After incubation, RNA/beads/antibody were washed twice in IP buffer, twice in low salt IP buffer (50 mM NaCl, 10 mM Tris–HCL, 0.1% NP‐40), and twice in high salt IP buffer (500 mM NaCl, 10 mM Tris–HCL, 0.1% NP‐40). RNA was eluted from beads with 25 μL of RLT buffer twice and elution was pooled and concentrated using Zymo RNA clean and concentrator kit‐5 (#R1015). Libraries were made using SMARTer Stranded Total RNA‐Seq Kit V4 without rRNA depletion (Takarabio, #634411) for IPed and input RNA, and sequenced using Illumina HiSeq X series with 40 M paired end reads (2 × 150 bp). Library preparation and sequencing was done by Admera Health. Three biological replicates per genotype and condition done with Synaptic Systems m^6^A antibody.

### 4.9 m^6^A enrichment analysis

Regions of m^6^A enrichment were found for each condition using MetPeak (v.1.1) (Cui et al., 2016) with default parameters, using the input and m^6^A pulldown bam files as input, and with the FlyBase FB2019_05 annotation provided. Peak locations (5' UTR, CDS, or 3' UTR) were defined from the regions indicated by MetPeak as having significant m^6^A enrichment. If a peak was not contained in one region (i.e., if the peak is partly in the CDS and partly in the 3' UTR), it was assigned to the region where more of the peak resided.

### 4.10 Differential m^6^A peak analysis

Regions of differential methylation between two conditions (frequently called “*Mettl3*‐dependent” or “m^6^A genes”) were found using RADAR (v.0.2.4) (Zhang et al., 2019) with input and m^6^A pulldown bam files as input, as well as the FlyBase FB2019_05 annotation. All replicates were used for differential peak calling. The minimum cutoff for bin filtering was 15, the cutoff was set as 0.05, and the Beta_cutoff was set as 0.5. Any region with an adjusted *p* < 0.05 was retained, and regions with a foldchange < −1 from the control (mCherry RNAi Control) to the knockout (*Mettl3* RNAi) or upregulation (UAS‐Aβ42) at 5d or 34d conditions. All genes were considered as all other genes expressed in the brain that did not have *Mettl3*‐dependent m^6^A.

### 4.11 m^6^A metaplot, heatmaps, and genome browser visualization

Heatmaps and metagene plots showing the location of m^6^A enrichment on a specific set of genes were constructed with using pheatmaps (v.1.0.12) and meRIPtools (v.0.2.1). Specifically, the exons of all transcripts in each gene were collapsed using the GenomicRanges (v.1.44.0) function reduce (Zhang, Luo, et al., 2020). Genes with a 5' UTR or 3' UTR shorter than 30 bp, a CDS shorter than 100 bp, or lacking a 5'/3' UTR (i.e., lncRNAs) were not considered in this analysis. For each gene, the 5' UTR and 3' UTR were tiled in 30 evenly spaced bins, and the CDS was tiled in 100 evenly spaced bins. The number of input and m^6^A reads overlapping each bin was calculated and this number was divided by the bin width and library size and a normalization factor of one million to produce a normalized reads per million in each bin. The heatmaps show the enrichment of m^6^A above input in each bin by dividing the m^6^A coverage by the input coverage after adding replicates from the same condition and sample type together. For heat map normalization, reads per million are normalized by the size of the bin, total reads, and library size. For genome browser snapshots, tracks visualized are log_2_ (m^6^A /input), or separated input and m^6^A‐IP tracks in supplementary figures. Tracks were made by first converting bam files to bigWig files using deepTools (v.3.5.1) (Ramírez et al., 2016) bamCoverage using CPM normalization, then deepTools bigwigCompare with operation log2.

### 4.12 GO and pathway analysis

GO analysis for genes was conducted using FlyMine (v.53) (Smith et al., 2012). The test correction was set to Holm‐Bonferroni with a max *p*‐value of 0.05. KEGG pathway analysis was done using the “enrichKEGG” function from ClusterProfiler (v.4.0.5) package in R (Yu et al., 2012). A list of all genes with detectable expression in the brain was used as background for both GO and pathway analysis.

### 4.13 FACS sorting

Brains (10–15 per replicate) from young (5 d) and older (34 d) males were dissected, collected into a tube with cold Schneider's medium (SM) containing 45 μM actinomycin D, and washed twice in PBS. Next, we added 300 μL of dissociating solution, which included papain diluted in PBS (Worthington PAP2, LK003178) and liberase (2.5 mg/mL). Brains were dissociated at 25°C using a shaker at 1000 rpm for 20 min and vigorous pipetting every 5 min, and passing through 25G 5/8 needle 7x. Dissociated tissue was filtered through 40 μm cell strainers and sorted on an BD FACS Aria II SORP with a 100 μm nozzle into a 96 well plate at the sorting facility of the University of Pennsylvania. Dead cells were excluded by staining with 4,6‐diamidino‐2phenylindole (DAPI). GFP+ cell gates were set according to the fluorescence profile of GFP‐ brain tissue, and nuclear stain Syto60. Desired glia or neuronal populations (~500 cells per replicate) were directly sorted into 10X lysis buffer (Takara 635013) plus RNAse inhibitor for library preparations using SMART‐seq v4. Library preps and sequencing was performed by Admera Health.

### 4.14 RNA‐seq analysis

Raw paired‐end fastqs were processed with TrimGalore (v.0.6.6) (https://github.com/FelixKrueger/TrimGalore) with default settings to remove Illumina adapters and mapped using STAR 2.7.3a (Dobin et al., 2013) to the *Drosophila melanogaster* genome annotation dm6. Unmapped and improperly paired reads were filtered out of aligned bam files. Reads per gene in the FlyBase release 2019_05 were computed using an R script using GenomicRanges (v.1.44.0) (Lawrence et al., 2013) summarize overlaps that counts the number of reads overlapping with the exons of each gene in the default “union” mode. Differential expression analysis was performed using DESeq2 (v.1.32.0) (Love et al., 2014), with count files produced by summarizeOverlaps as input. PCA plots were made using the plotPCA function in DESeq2, with variance stabilized counts as the input. MA plots were constructed from the adjusted *p*‐values and baseMean values output from DESeq2, and volcano plots were constructed from adjusted *p*‐values and fold changes reported by DESeq2. Normalized counts produced by DESeq2 were used to show expression levels. For FACS RNA‐seq analysis, we utilized RUVseq (v.3.17), and samples were processed utilizing RUVr approach for normalization of read counts and PCA plot analysis. Differential expression analysis was done using DESeq2 and differentially expressed genes were considered to be any gene with a *p*_adj_ < 0.05.

### 4.15 Quantification and statistical analysis

Statistical tests used were performed on GraphPad Prism (v.9), and are indicated in the figure legend. *p* < 0.05 was considered significant. Unpaired two‐tailed *t*‐tests were used when comparing two groups; One‐way ANOVA was used when comparing multiple groups followed by Tukey's post‐test when each group was compared against every other group, Sidak's posttest when pre‐defined groups were compared to each other, or Dunnett's test when comparing to a defined control sample. Two‐way ANOVA was used when there were two factors in the analysis.

### 4.16 TRAP assay

TRAP assay sample preparation was done using a protocol similar to (Chen & Dickman, 2017). Fly brains (30 brains/sample) were dissected and stored in buffer (10 mM Hepes, 150 mM KCl, 5 mM MgCl2, 100ug/ml Cyclohexamide, 1x protease inhibitor, Rnase inhibitor) in −80°C until samples were ready to process. Protein G Magnetic beads were coated with 4 μg of anti‐flag antibody, and resuspended in reaction buffer (10 mM Hepes, 150 mM KCl, 5 mM MgCl2, 100ug/ml Cyclohex, 1x protease inhibitor (Sigma, #11873580001), + 0.1% Triton X‐100, RNase inhibitor) (Thermo Fisher, #AM2696). Samples with brain tissue were homogenized in lysis buffer (reaction buffer +0.5% triton‐x). 20% of sample was saved as input control and RNA extraction using Zymo clean and concentrator kit‐5. The rest of the sample was added to antibody plus beads, and incubated for 4 h at 4°C. Beads + RNA was washed and RNA was extracted from beads using zymo clean and concentrator kit −5. Libraries were made using SMARTer Stranded Total RNASeq Kit V4 for IPed and input RNA, and sequenced using Illumina HiSeq X series with 40 M paired end reads (2 × 150 bp). Library preparation and sequencing was done by Admera Health. Three biological replicates per genotype and condition were done. Translation efficiency was calculated by log_2_ value of rpkm IP divided by rpkm of input.

## AUTHOR CONTRIBUTIONS

Alexandra E. Perlegos conceived, designed, and performed experiments, statistical analysis, bioinformatic analysis, and analyzed data. China N. Byrns performed and assisted in FACS sorting experiments and design. Nancy M. Bonini conceived, designed experiments, analyzed data, and supervised the research. Alexandra E. Perlegos and Nancy M. Bonini wrote the manuscript.

## CONFLICT OF INTEREST STATEMENT

Authors declare no competing interests.

## Supporting information


Appendix S1:

Appendix S2:

Appendix S3:

Appendix S4:

Supplementary Figures S1–S6

## Data Availability

The Raw sequencing data generated in this study have been deposited in the Gene Expression Omnibus GSE235989. Any additional inquiries can be directed to the corresponding author.
