# Supplementary material for: Cell type‐specific regulation of m6A modified RNAs in the aging Drosophila brain
Source: Aging Cell. 2024 Jan 11;23(3):e14076. doi: 10.1111/acel.14076 (PMC10928574; doi:10.1111/acel.14076)
Supplement: Supplementary file 5 — Supplementary Figures S1–S6 [file ACEL-23-e14076-s002.pdf]

Supplementary Materials for

Cell type specific regulation of m^{6}A modified RNAs in the aging *Drosophila* brain

Alexandra E. Perlegos [1,2], China N. Byrns [1,2,3], Nancy M. Bonini[1,2*]

[1]Neuroscience Graduate Group, University of Pennsylvania, Philadelphia, PA, 19104, USA.
[2]Department of Biology, University of Pennsylvania, Philadelphia, PA, 19104, USA.
[3]Medical Scientist Training Program, University of Pennsylvania, Philadelphia, PA, 19104, USA.

This PDF includes:

Supplementary figures 1-6

Other supporting materials include the following:

Supplementary Data Files:
Supplementary Data 1 – *Drosophila* Lines and Primers
Supplementary Data 2 – m^{6}A RADAR files
Supplementary Data 3 – GO terms and KEGG Pathway
Supplementary Data 4 – RNA-seq differential expression files

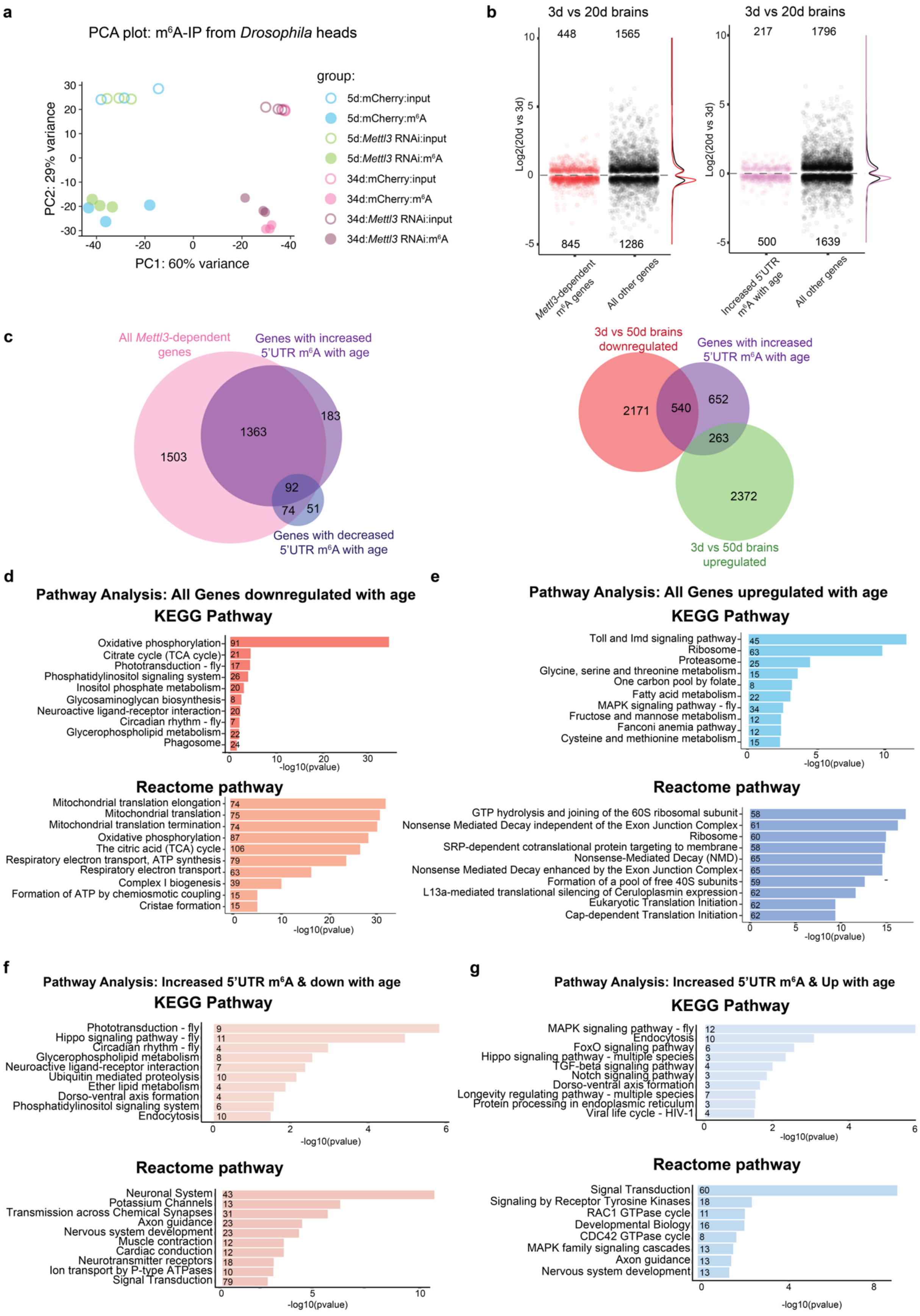
a
PCA plot: m6A-IP from Drosophila heads
group:
5d:mCherry:input
5d:mCherry:m6A
5d:Mettl3 RNAi:input
5d:Mettl3 RNAi:m6A
34d:mCherry:input
34d:mCherry:m6A
34d:Mettl3 RNAi:input
34d:Mettl3 RNAi:m6A
PC1: 60% variance
PC2: 29% variance
b
3d vs 20d brains
3d vs 20d brains
448
1565
845
1286
217
1796
500
1639
Log2(20d vs 3d)
Mettl3-dependent m6A genes
All other genes
Increased 5'UTR m6A with age
All other genes
c
All Mettl3-dependent genes
Genes with increased 5'UTR m6A with age
1503
1363
183
92
74
51
Genes with decreased 5'UTR m6A with age
3d vs 50d brains downregulated
Genes with increased 5'UTR m6A with age
2171
540
652
263
2372
3d vs 50d brains upregulated
d
Pathway Analysis: All Genes downregulated with age
KEGG Pathway
Oxidative phosphorylation
Citrate cycle (TCA cycle)
Phototransduction - fly
Phosphatidylinositol signaling system
Inositol phosphate metabolism
Glycosaminoglycan biosynthesis
Neuroactive ligand-receptor interaction
Circadian rhythm - fly
Glycerophospholipid metabolism
Phagosome
-log10(pvalue)
Reactome pathway
Mitochondrial translation elongation
Mitochondrial translation
Mitochondrial translation termination
Oxidative phosphorylation
The citric acid (TCA) cycle
Respiratory electron transport, ATP synthesis
Respiratory electron transport
Complex I biogenesis
Formation of ATP by chemiosmotic coupling
Cristae formation
-log10(pvalue)
e
Pathway Analysis: All Genes upregulated with age
KEGG Pathway
Toll and Imd signaling pathway
Ribosome
Proteasome
Glycine, serine and threonine metabolism
One carbon pool by folate
Fatty acid metabolism
MAPK signaling pathway - fly
Fructose and mannose metabolism
Fanconi anemia pathway
Cysteine and methionine metabolism
-log10(pvalue)
Reactome pathway
GTP hydrolysis and joining of the 60S ribosomal subunit
Nonsense Mediated Decay independent of the Exon Junction Complex
Ribosome
SRP-dependent cotranslational protein targeting to membrane
Nonsense-Mediated Decay (NMD)
Nonsense Mediated Decay enhanced by the Exon Junction Complex
Formation of a pool of free 40S subunits
L13a-mediated translational silencing of Ceruloplasmin expression
Eukaryotic Translation Initiation
Cap-dependent Translation Initiation
-log10(pvalue)
f
Pathway Analysis: Increased 5'UTR m6A & down with age
KEGG Pathway
Phototransduction - fly
Hippo signaling pathway - fly
Circadian rhythm - fly
Glycerophospholipid metabolism
Neuroactive ligand-receptor interaction
Ubiquitin mediated proteolysis
Ether lipid metabolism
Dorso-ventral axis formation
Phosphatidylinositol signaling system
Endocytosis
-log10(pvalue)
Reactome pathway
Neuronal System
Potassium Channels
Transmission across Chemical Synapses
Axon guidance
Nervous system development
Muscle contraction
Cardiac conduction
Neurotransmitter receptors
Ion transport by P-type ATPases
Signal Transduction
-log10(pvalue)
g
Pathway Analysis: Increased 5'UTR m6A & Up with age
KEGG Pathway
MAPK signaling pathway - fly
Endocytosis
FoxO signaling pathway
Hippo signaling pathway - multiple species
TGF-beta signaling pathway
Notch signaling pathway
Dorso-ventral axis formation
Longevity regulating pathway - multiple species
Protein processing in endoplasmic reticulum
Viral life cycle - HIV-1
-log10(pvalue)
Reactome pathway
Signal Transduction
Signaling by Receptor Tyrosine Kinases
RAC1 GTPase cycle
Developmental Biology
CDC42 GTPase cycle
MAPK family signaling cascades
Axon guidance
Nervous system development
-log10(pvalue)

Supplementary Figure 1: m^{6}A-IP sequencing in the aging brain

- **a.** Principal component analysis (PCA) plot of m^{6}A-IP-sequencing experiment using control and *Mettl3* heads at 5d and 34d. Each circle is one biological replicate. PC1 separates based on age (5d vs 34d) whereas PC2 separates based on Input versus m^{6}A-IP.
- **b.** Plot of significantly differentially expressed genes padj<0.05 of control (w^{1118}) brains with age (20d vs 3d). Positive logFC indicates an increase in transcript level with age. *Mettl3*-dependent m^{6}A transcripts (red), all other non-m^{6}A transcripts (black), transcripts with increased 5'UTR m^{6}A with age (purple).
- **c.** Left, Venn diagram of *Mettl3*-dependent genes (5d and 34d) overlapped with genes with increased 5'UTR m^{6}A with age and genes with decreased 5'UTR m^{6}A with age. Right, Venn diagram overlap of genes with increased 5'UTR m^{6}A with age, genes downregulated with age (50d vs 3d) and genes upregulated with age.
- **d.** Pathway analysis of all genes downregulated with age in the brain (50d vs 3d).
- **e.** Pathway analysis of all genes upregulated with age in the brain (50d vs 3d).
- **f.** Pathway analysis of genes with increased 5'UTR m^{6}A with age that are downregulated with age in the brains (50d vs 3d RNA-seq).
- **g.** Pathway analysis of genes with increased 5'UTR m^{6}A with age that are upregulated with age in the brain (50d vs 3d RNA-seq).

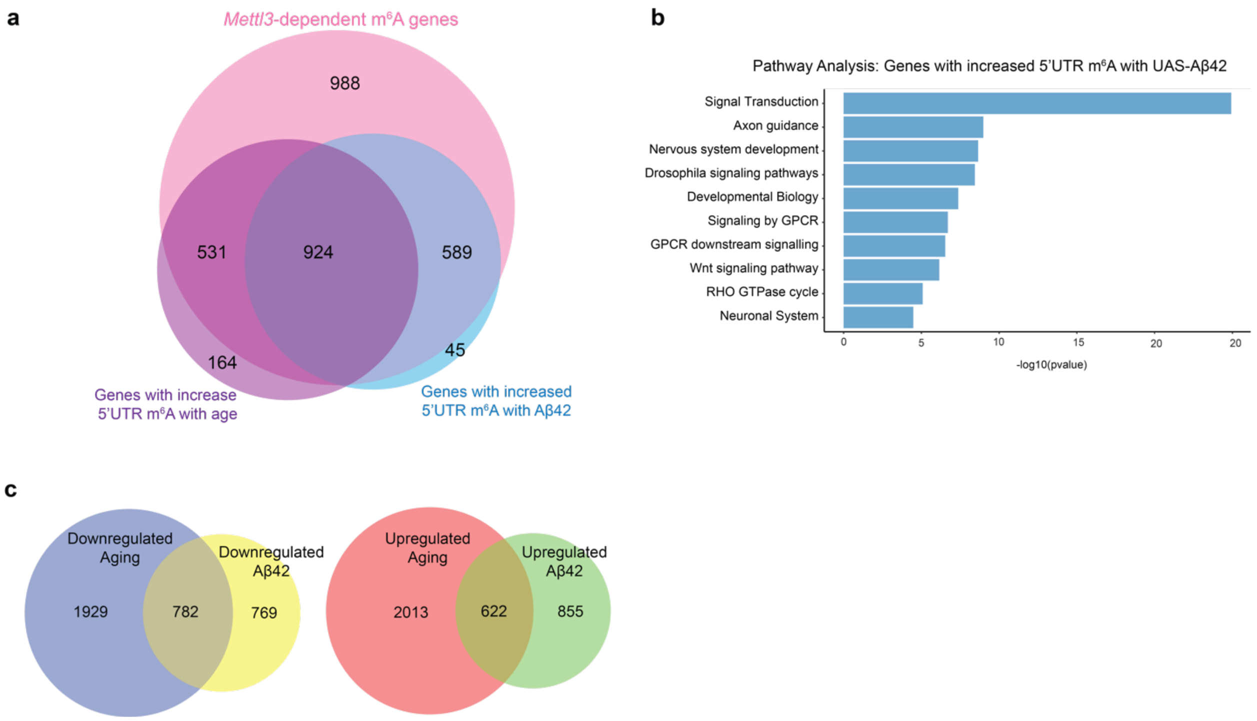

Supplementary Figure 2: m^{6}A modulation with Aβ42

- **a.** Venn diagram of *Mettl3*-dependent genes (5d and 34d) overlap with genes with increased 5'UTR m^{6}A with age and genes with increased 5'UTR m^{6}A with UAS-Aβ42.
- **b.** Reactome pathway analysis of genes with increased 5'UTR m^{6}A in UAS-Aβ42 condition.
- **c.** Comparison overlaps of transcripts upregulated or downregulated with age or Aβ42 expression in neurons of the brain.

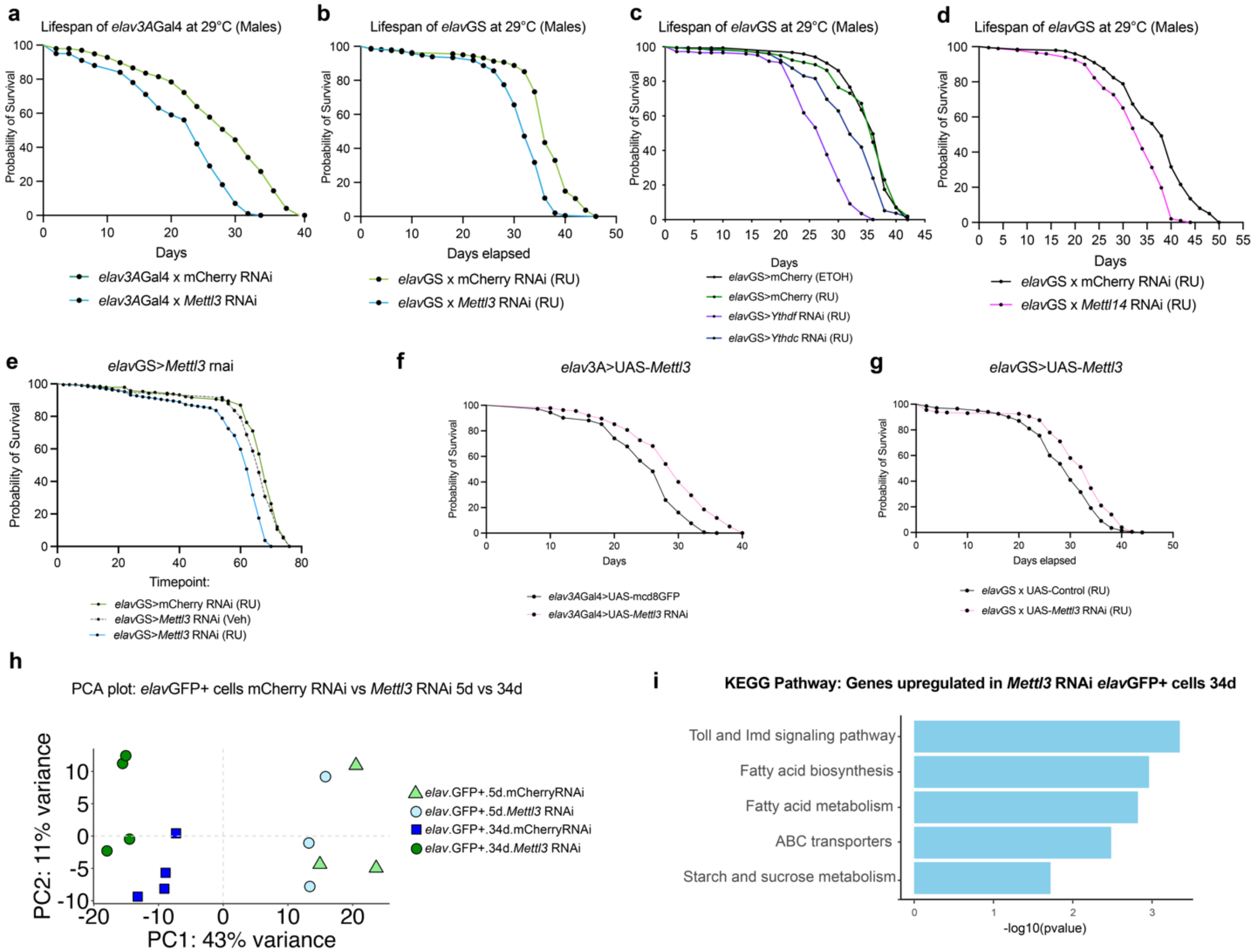
a
Lifespan of elav3AGal4 at 29°C (Males)
Probability of Survival
Days
elav3AGal4 x mCherry RNAi
elav3AGal4 x Mettl3 RNAi
b
Lifespan of elavGS at 29°C (Males)
Probability of Survival
Days elapsed
elavGS x mCherry RNAi (RU)
elavGS x Mettl3 RNAi (RU)
c
Lifespan of elavGS at 29°C (Males)
Probability of Survival
Days
elavGS>mCherry (ETOH)
elavGS>mCherry (RU)
elavGS>Ythdf RNAi (RU)
elavGS>Ythdc RNAi (RU)
d
Lifespan of elavGS at 29°C (Males)
Probability of Survival
Days
elavGS x mCherry RNAi (RU)
elavGS x Mettl14 RNAi (RU)
e
elavGS>Mettl3 rnai
Probability of Survival
Timepoint:
elavGS>mCherry RNAi (RU)
elavGS>Mettl3 RNAi (Veh)
elavGS>Mettl3 RNAi (RU)
f
elav3A>UAS-Mettl3
Probability of Survival
Days
elav3AGal4>UAS-mcd8GFP
elav3AGal4>UAS-Mettl3 RNAi
g
elavGS>UAS-Mettl3
Probability of Survival
Days elapsed
elavGS x UAS-Control (RU)
elavGS x UAS-Mettl3 RNAi (RU)
h
PCA plot: elavGFP+ cells mCherry RNAi vs Mettl3 RNAi 5d vs 34d
PC2: 11% variance
PC1: 43% variance
elav.GFP+.5d.mCherryRNAi
elav.GFP+.5d.Mettl3 RNAi
elav.GFP+.34d.mCherryRNAi
elav.GFP+.34d.Mettl3 RNAi
i
KEGG Pathway: Genes upregulated in Mettl3 RNAi elavGFP+ cells 34d
Toll and Imd signaling pathway
Fatty acid biosynthesis
Fatty acid metabolism
ABC transporters
Starch and sucrose metabolism
-log10(pvalue)

Supplementary Figure 3: m^{6}A modulation in Neurons

- **a.** Lifespan of animals expressing control RNAi or *Mettl3* RNAi in neurons. (*elav3A*Gal4>mCherry RNAi vs *elav3A*Gal4>*Mettl3* RNAi). 29°C, n=100, n=100, p****<0.0001, Log-rank test.
- **b.** Lifespan of animals expressing control RNAi or *Mettl3* RNAi in neurons. Animals put on RU486 food as adults 1-2d post eclosion. (*elav*GS-Gal4>mCherry RNAi vs *elav*GS-Gal4>*Mettl3* RNAi). 29°C, n=180, n=161, p****<0.0001, Log-rank test.
- **c.** Lifespan of animals expressing control RNAi, *Ythdc1* RNAi or *Ythdf* RNAi in neurons. Animals put on RU486 food as adults 1-2d post eclosion. (*elav*GS-Gal4>mCherry RNAi; *elav*GS-Gal4>*Ythdf* RNAi; *elav*GS-Gal4>*Ythdc1* RNAi) 29°C, n=140 (ETOH), n=180, n=180, n=180 p****<0.0001, Log-rank test. Control ETOH lifespan is not significantly different from control lifespan on RU486 food.
- **d.** Lifespan of animals expressing control RNAi or *Mettl14* RNAi in neurons. Animals put on RU486 food as adults 1-2d post eclosion. (*elav*GS-Gal4>mCherry RNAi vs *elav*GS-Gal4>*Mettl14* RNAi). 29°C, n=190, n=190, p****<0.0001, Log-rank test.
- **e.** Lifespan of animals expressing control RNAi or *Mettl3* RNAi in neurons, animals put on RU486 or Vehicle food as adults 1-2d post eclosion. (*elav*GS-Gal4>mCherry RNAi vs *elav*GS-Gal4>*Mettl3* RNAi Vehicle or RU486 food). 25°C, n=190, n=190, n=190, p****<0.0001 (mCherry RNAi vs *Mettl3* RU), p****<0.0001 (*Mettl3* Veh vs *Mettl3* RU), Log-rank test.
- **f.** Lifespan of animals expressing control or upregulation of *Mettl3* in neurons. (*elav3A*Gal4>UAS-mCD8GFP vs *elav3A*Gal4>UAS-*Mettl3*). Lifespans performed at 29°C, n=140, n=140, p****<0.0001, Log-rank test.
- **g.** Lifespan of animals expressing control or upregulation of *Mettl3* in neurons only as adults. (*elav*GS>UAS-mCD8GFP vs *elav*GS>UAS-*Mettl3*). Animals put on RU486 food as adults 1-2d post eclosion. Lifespans performed at 29°C, n=200, n=200, p****<0.0001, Log-rank test.
- **h.** PCA plot of FACS sorting RNA sequencing of *elav*GFP+ cell samples.
- **i.** KEGG analysis of genes upregulated in *Mettl3* RNAi *elav*GFP+ cells vs mCherry RNAi cells at 34d.

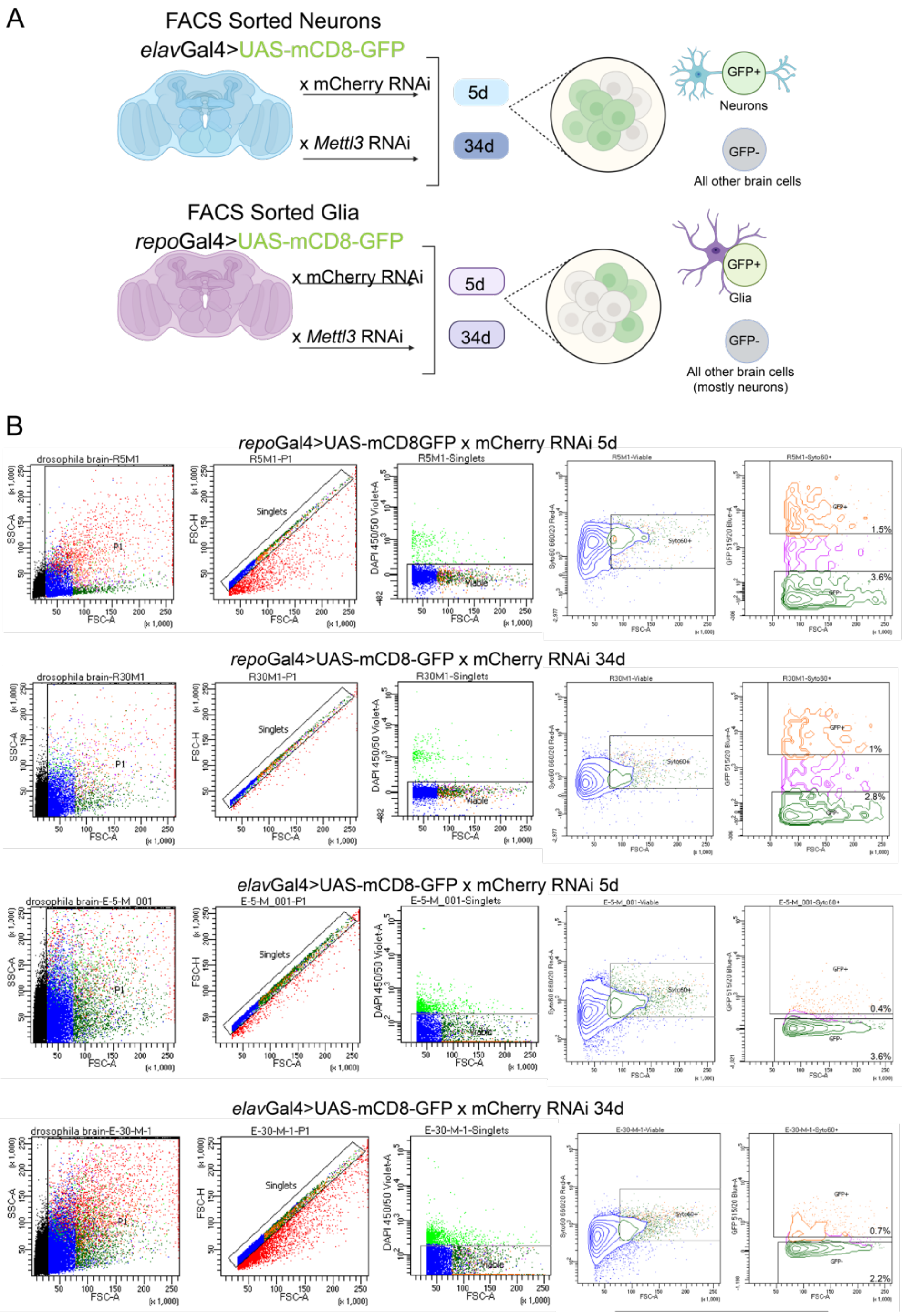

Supplementary Figure 4: FACS sorting of *Drosophila* Neurons and Glial cells for RNA-sequencing.

a. Schematic of FACS sorting of *elav*GFP+ or repoGFP+ positive cells with or without *Mettl3* RNAi. Cells sorted from 5d or 34d brains for each genotype.
b. Gating strategy examples from each experimental condition for FACS.

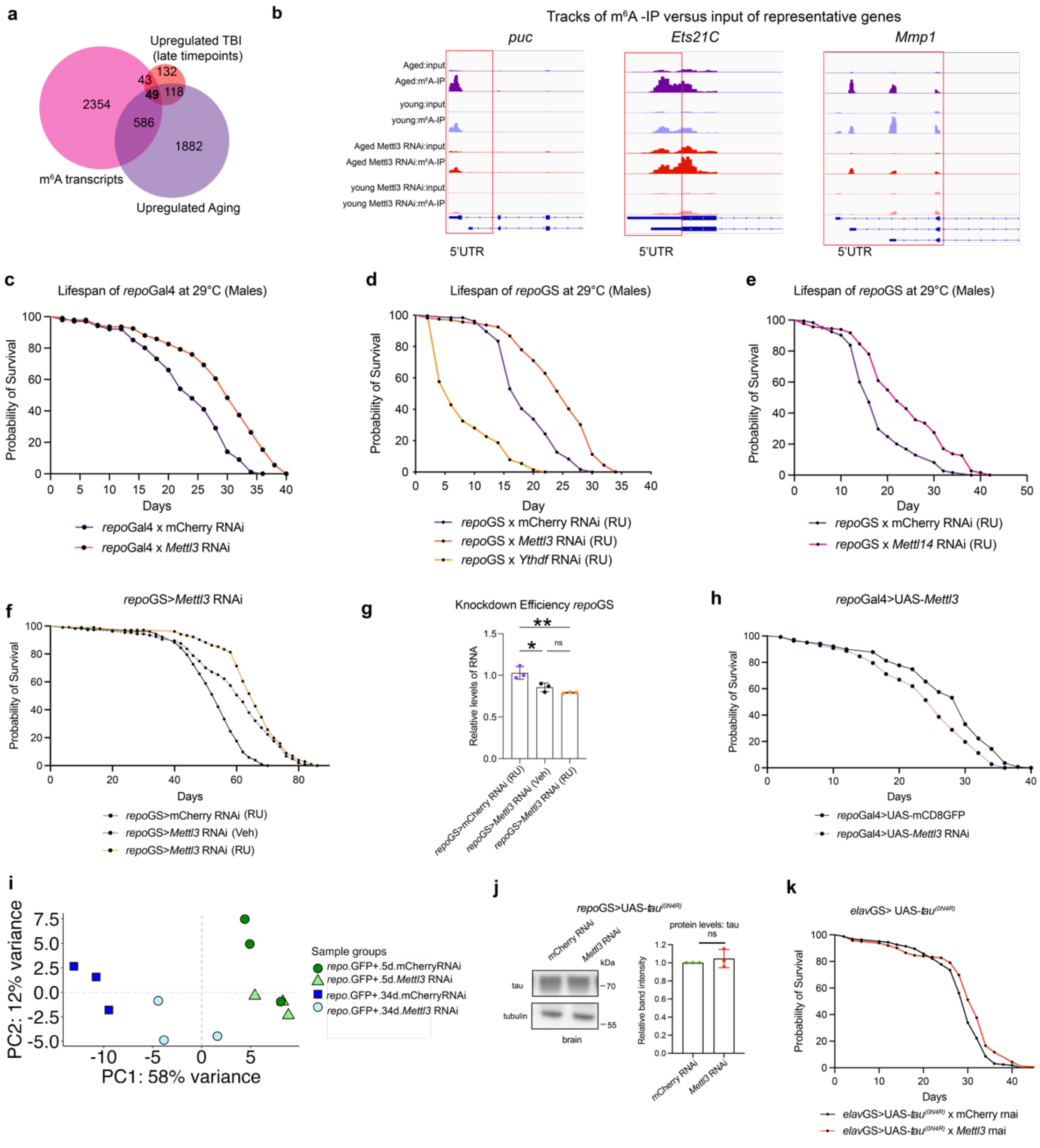

a
Upregulated TBI (late timepoints)
132
43
49
118
2354
586
1882
m6A transcripts
Upregulated Aging
b
Tracks of m6A -IP versus input of representative genes
puc
Ets21C
Mmp1
Aged:input
Aged:m6A-IP
young:input
young:m6A-IP
Aged Mettl3 RNAi:input
Aged Mettl3 RNAi:m6A-IP
young Mettl3 RNAi:input
young Mettl3 RNAi:m6A-IP
5'UTR
c
Lifespan of repoGal4 at 29°C (Males)
Probability of Survival
Days
repoGal4 x mCherry RNAi
repoGal4 x Mettl3 RNAi
d
Lifespan of repoGS at 29°C (Males)
Probability of Survival
Day
repoGS x mCherry RNAi (RU)
repoGS x Mettl3 RNAi (RU)
repoGS x Ythdf RNAi (RU)
e
Lifespan of repoGS at 29°C (Males)
Probability of Survival
Day
repoGS x mCherry RNAi (RU)
repoGS x Mettl14 RNAi (RU)
f
repoGS>Mettl3 RNAi
Probability of Survival
Days
repoGS>mCherry RNAi (RU)
repoGS>Mettl3 RNAi (Veh)
repoGS>Mettl3 RNAi (RU)
g
Knockdown Efficiency repoGS
Relative levels of RNA
**
*
ns
repoGS>mCherry RNAi (RU)
repoGS>Mettl3 RNAi (Veh)
repoGS>Mettl3 RNAi (RU)
h
repoGal4>UAS-Mettl3
Probability of Survival
Days
repoGal4>UAS-mCD8GFP
repoGal4>UAS-Mettl3 RNAi
i
PC2: 12% variance
PC1: 58% variance
Sample groups
repo.GFP+.5d.mCherryRNAi
repo.GFP+.5d.Mettl3 RNAi
repo.GFP+.34d.mCherryRNAi
repo.GFP+.34d.Mettl3 RNAi
j
repoGS>UAS-tau(0N4R)
mCherry RNAi
Mettl3 RNAi
kDa
tau
tubulin
70
55
brain
protein levels: tau
ns
Relative band intensity
mCherry RNAi
Mettl3 RNAi
k
elavGS> UAS-tau(0N4R)
Probability of Survival
Days
elavGS>UAS-tau(0N4R) x mCherry rnai
elavGS>UAS-tau(0N4R) x Mettl3 rnai

Supplementary Figure 5: m^{6}A modulation in Glia

- **a.** Comparison of transcripts upregulated in the brain with age, upregulated in head in late timepoints of traumatic brain injury (TBI) (3,7,10,15 d, head tissue)[54] and m^{6}A marked transcripts with aging.
- **b.** Example transcripts that are upregulated in TBI and with aging, and show increased m^{6}A modification with age.
- **c.** Lifespan curve of animals expressing control RNAi or *Mettl3* RNAi in glia (*repo*Gal4>mCherry RNAi vs *repo*Gal4>*Mettl3* RNAi). Lifespan performed at 29°C, n=94, n=100, p****<0.0001, Log-rank test.
- **d.** Lifespan curve of animals expressing control RNAi, *Mettl3* RNAi, or *Ythdf* RNAi in glia as adults (*repo*GS-Gal4>mCherry RNAi vs *repo*GS-Gal4>*Mettl3* RNAi vs *repo*GS-Gal4>*Ythdf* RNAi). Animals put on RU486 food 1-2d post eclosion. Lifespans performed at 29°C, n=180, n=170, n=180, p****<0.0001, Log-rank test.
- **e.** Lifespan curve of animals expressing control RNAi or *Mettl14* RNAi in glia, *repo*GS. (*repo*GS-Gal4>mCherry RNAi vs *repo*GS-Gal4>*Mettl14* RNAi). Animals put on RU486 food as adults 1-2 d post eclosion. Lifespans performed at 29°C, n=180, n=160, p****<0.0001, Log-rank test.
- **f.** Lifespan curve of animals expressing control RNAi or *Mettl3* RNAi in glia (*repo*GS-Gal4>mCherry RNAi vs *repo*GS-Gal4>*Mettl3* RNAi on vehicle or RU486 food). Animals put on RU486 food as adults 1-2 d post eclosion. Lifespans performed at 25°C, n=103, n=103, n=103, p****<0.0001 (mCherry RU vs *Mettl3* RU), p****<0.0001 (mCherry RU vs *Mettl3* Veh), p*<0.05 (*Mettl3* RU vs *Mettl3* Veh), Log-rank test.
- **g.** Knockdown validation of *Mettl3* RNAi in *repo*GS background after 6 d on RU486 or vehicle food. Animals put on RU486 or vehicle food as adults. p**<0.01, p*<0.05, ns= not significant, One-way ANOVA.
- **h.** Lifespan curve of animals expressing control or upregulation of *Mettl3* in glia, *repo*Gal4. (*repo*Gal4>UAS-mCD8GFP vs *repo*Gal4>UAS-*Mettl3*). Lifespans performed at 29°C, n=140, n=140, p****<0.0001, Log-rank test.
- **i.** PCA analysis of FACS sorted *repo*GFP+ cell samples 5d vs 34d RNA-sequencing.
- **j.** Levels of total tau protein in the brain with *repo*GS>0N4R x mCherry RNAi or *Mettl3* RNAi. Tubulin used as loading control. n=10 brains per replicate, 3 biological replicates, p=0.4667, Student's t-test, ns = not significant.
- **k.** Lifespan animals expressing human wild type *tau*$^{(0N4R)}$ in neurons with control mCherry RNAi, or *Mettl3* RNAi. (*elav*GS-Gal4>UAS-*tau*$^{(0N4R)}$ x mCherry RNAi vs *elav*GS-Gal4>UAS-*tau*$^{(0N4R)}$ x *Mettl3* RNAi). Animals put on RU486 food as adults 1-2d post eclosion. Lifespan performed at 29°C, n=162, n=162, p***<0.0006, Log-rank test.

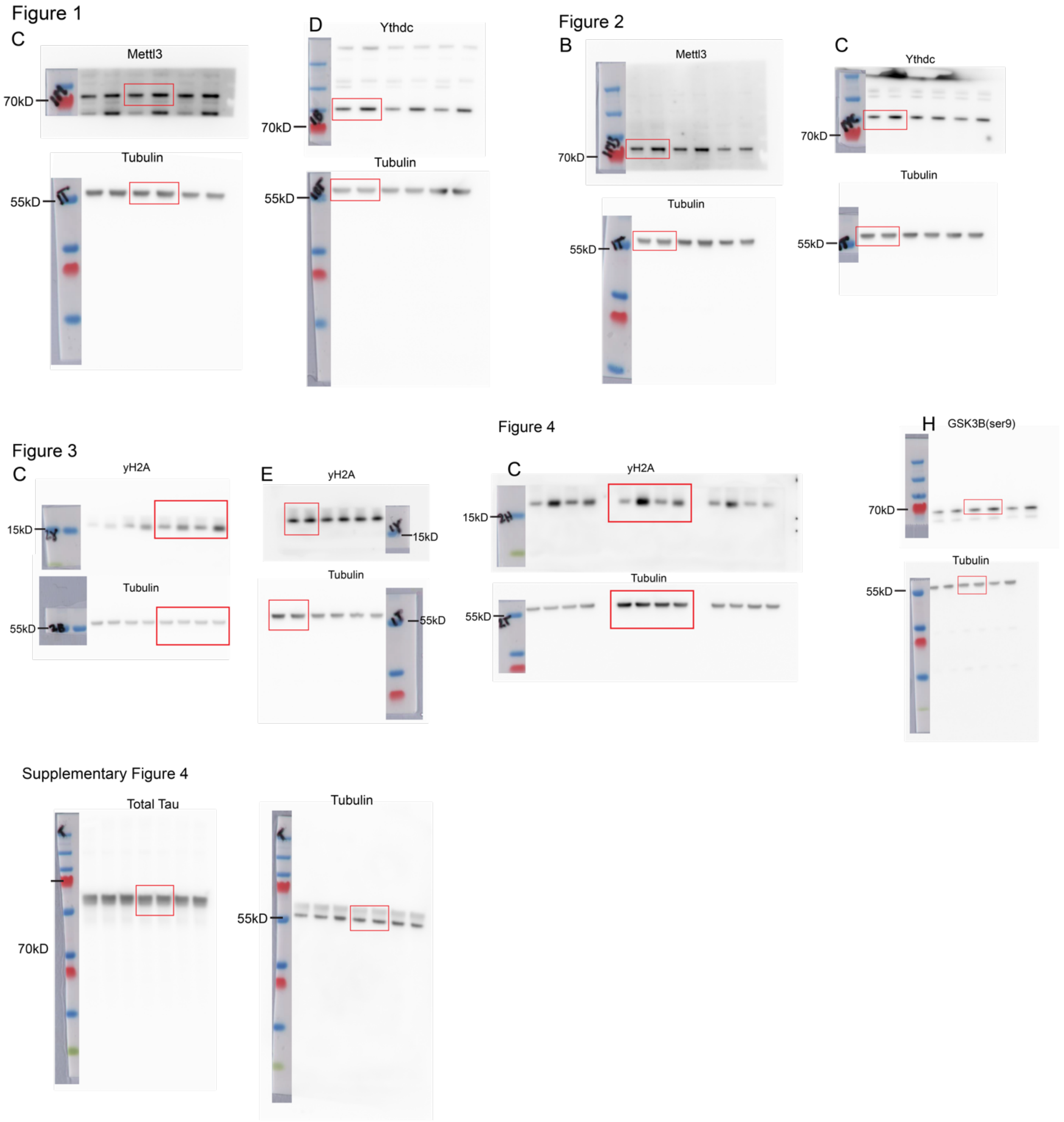

Supplementary Figure 6. Uncropped lanes for all western immunoblots.
Each blot is labeled for its figure panel. The red box indicate the representative lanes used in each figure panel. Page Ruler Plus Prestained Protein Ladder (Thermo Scientific, 22619).
